# Proteasome localization and activity in pig brain and *in vivo* small molecule screening for activators

**DOI:** 10.3389/fncel.2024.1353542

**Published:** 2024-02-26

**Authors:** Adriana Amrein Almira, May W. Chen, Nagat El Demerdash, Cameron Javdan, Dongseok Park, Jennifer K. Lee, Lee J. Martin

**Affiliations:** ^1^Department of Anesthesiology and Critical Care Medicine, Johns Hopkins University School of Medicine, Baltimore, MD, United States; ^2^Departments of Pediatrics, Johns Hopkins University School of Medicine, Baltimore, MD, United States; ^3^Department of Pathology, Johns Hopkins University School of Medicine, Baltimore, MD, United States; ^4^Department of Neuroscience, Johns Hopkins University School of Medicine, Baltimore, MD, United States; ^5^Pathobiology Graduate Training Program, Johns Hopkins University School of Medicine, Baltimore, MD, United States

**Keywords:** aging, chlorpromazine, encephalopathy, neonatal brain injury, protein aggregation, proteinopathy, proteasome nuclear satellite, pyrazolone

## Abstract

**Introduction:**

Loss of proteasome function, proteinopathy, and proteotoxicity may cause neurodegeneration across the human lifespan in several forms of brain injury and disease. Drugs that activate brain proteasomes *in vivo* could thus have a broad therapeutic impact in neurology.

**Methods:**

Using pigs, a clinically relevant large animal with a functionally compartmental gyrencephalic cerebral cortex, we evaluated the localization and biochemical activity of brain proteasomes and tested the ability of small molecules to activate brain proteasomes.

**Results:**

By Western blotting, proteasome protein subunit PSMB5 and PSMA3 levels were similar in different pig brain regions. Immunohistochemistry for PSMB5 showed localization in the cytoplasm (diffuse and particulate) and nucleus (cytoplasm < nucleus). Some PSMB5 immunoreactivity was colocalized with mitochondrial (voltage-gated anion channel and cyclophilin D) and cell death (Aven) proteins in the neuronal soma and neuropil in the neocortex of pig and human brains. In the nucleus, PSMB5 immunoreactivity was diffuse, particulate, and clustered, including perinucleolar decorations. By fluorogenic assay, proteasome chymotrypsin-like activities (CTL) in crude tissue soluble fractions were generally similar within eight different pig brain regions. Proteasome CTL activity in the hippocampus was correlated with activity in nasal mucosa biopsies. In pilot analyses of subcellular fractions of pig cerebral cortex, proteasome CTL activity was highest in the cytosol and then ~50% lower in nuclear fractions; ~15–20% of total CTL activity was in pure mitochondrial fractions. With in-gel activity assay, 26S-singly and -doubly capped proteasomes were the dominant forms in the pig cerebral cortex. With a novel *in situ* histochemical activity assay, MG132-inhibitable proteasome CTL activity was localized to the neuropil, as a mosaic, and to cell bodies, nuclei, and centrosome-like perinuclear satellites. In piglets treated intravenously with pyrazolone derivative and chlorpromazine over 24 h, brain proteasome CTL activity was modestly increased.

**Discussion:**

This study shows that the proteasome in the pig brain has relative regional uniformity, prominent nuclear and perinuclear presence with catalytic activity, a mitochondrial association with activity, 26S-single cap dominance, and indications from small molecule systemic administration of pyrazolone derivative and chlorpromazine that brain proteasome function appears safely activable.

## Introduction

The pig (*Sus domesticus*) has an increasing presence in neurobiology, behavioral neurology, and experimental neuropathology (Lind et al., 2007; Kornum and Knudsen, 2011; Koehler et al., 2018). Transgenic pigs harboring human mutant genes have been generated to model amyotrophic lateral sclerosis (Yang et al., 2014), spinal muscular atrophy (Lorson et al., 2011), and ataxia telangiectasia (Beraldi et al., 2015). These swine models are generally robust in displaying neurological and neuropathological phenotypes like human disease and, in many instances, have a more faithful representation than the rodent counterparts. Pigs also make superb models of acute acquired brain injury, including traumatic (Dai et al., 2018; Wang et al., 2023) and global hypoxic–ischemic (Martin et al., 1997; Koehler et al., 2018; Primiani et al., 2023), with the latter showing patterns of selective vulnerability very similar to human infants (Johnston, 1998). Through cardiovascular and cerebrovascular monitoring, pigs show clinically relevant pathophysiology (Brambrink et al., 1999; Primiani et al., 2023; Wang et al., 2023). As a gyrencephalic large animal, their brain neuroanatomy has many similarities in structure and disease vulnerability (Martin et al., 1997; Yang et al., 2014; Koehler et al., 2018; Yan et al., 2018), and pigs purportedly have brain resting-state networks homologous to humans (Simchick et al., 2019). Pigs were used recently to define, in the gyrencephalic brain, the cellular basis for unexplained signal intensity changes that are seen in clinical magnetic resonance imaging of infants with encephalopathy (Lee et al., 2020, 2021), and they were used to study hypothermic protection of functionally different neocortical regions after neonatal hypoxia–ischemia (Primiani et al., 2023). These attributes consolidate the pig as an ideal system for experimental cellular and molecular neuropathology, disease mechanism identification, therapeutic target realization, and therapeutic drug testing.

The proteasome is one hopeful mechanism-based therapeutic target to lessen human neurodegeneration (Chen et al., 2012; Trippier et al., 2014; Zhang et al., 2015; Jones et al., 2017; Trader et al., 2017; Santoro et al., 2020). This organelle functions in proteostasis. Proteasomes are large multisubunit proteins and multicatalytic proteinase complexes that constitute the major machinery for non-lysosomal protein degradation in eukaryotic cells (Hershko et al., 1980; Glickman and Ciechanover, 2002). Proteins that are damaged, misfolded, mutated, or senescent and targeted for turnover are degraded by the proteasome (Hershko et al., 1980; Glickman and Ciechanover, 2002). Aberrant proteostasis is a putative fundamental mechanism of brain aging, injury, and disease (Keller et al., 2000; Davidson and Pickering, 2023). Proteotoxicity due to proteasome dysfunction is implicated in several age-related human neurological disorders involving superoxide dismutase-1 in mutant and oxidized wild-type forms (Kabashi et al., 2004; Tashiro et al., 2012; Rotunno and Bosco, 2013; Kim et al., 2020), TAR-DNA binding protein-43 (Watanabe et al., 2013), and α-synuclein (McNaught and Jenner, 2001; Wong and Krainc, 2017; Kumar et al., 2018). Oligomerization and aggregation proteins may have key roles in the prion-like origin and connectome-wide spreading of nervous system disease (Esiri et al., 1990; Pearson, 1996; Cushman et al., 2010). Originally deemed to be a driver only in adult age-related chronic neurodegenerative disease (Davidson and Pickering, 2023), proteinopathy now appears to have roles in acute brain injury in the neonatal period (Lee et al., 2016; Santos et al., 2018; Martin et al., 2019). Protein oxidation evolves rapidly in concert with α-synuclein oligomerization in a neonatal mouse model of traumatic brain injury (Martin et al., 2019). Aberrant brain proteasome localization and activity, increased protein oxidative damage (carbonyl accumulation), and elevated protein ubiquitination were discovered in neonatal hypoxic–ischemic piglets (Santos et al., 2018; El Demerdash et al., 2021). These studies seed the idea that proteinopathy as a pathological mechanism in the nervous system is not limited to aging and neurodegenerative disease in adulthood (Martin et al., 2019, 2022); thus, the clinical need and application of targeting the proteasome for activation may broaden.

Experiments on the proteasome in pigs can satisfy important preclinical needs to advance the field of nervous system therapeutics for injury and disease. Prior studies suggest that species differences exist in the localization of proteasome subunits (Adori et al., 2006) and the biochemical activities (Sesma et al., 2003; Pride et al., 2015; El Demerdash et al., 2021). Information on proteasome localization and activity in the swine brain is limited (Santos et al., 2018; El Demerdash et al., 2021). It is important to examine proteasome localizations and activities in animal alternatives to rodents to better appreciate the attributes and relevance of models used for brain injury and disease, particularly those involving proteinopathy, and to develop proteasome-targeted therapeutics. Pig neurons have molecular degeneration mechanisms that appear to be more like human neurons than mouse neurons are to human neurons (Yang et al., 2014; Martin and Chang, 2018). In addition, a step forward would be the identification of CNS-acting small molecule activators of the proteasome that have safe and realistic translational applications, such as systemic administration. In this regard, a single study showed that a small molecule derivative of pyrazolone with *in vitro* proteasome agonist activity extends the lifespan of a mouse model of ALS, but the corresponding activation of the brain proteasome was not examined, so the mechanism of the lifespan extension is uncertain (Chen et al., 2012). Here, we address several aspects of proteasome localization and function in pig brain and provide a pilot *in vivo* screening of two drugs (a derivative of pyrazolone and chlorpromazine) previously shown to activate the proteasome in cell culture (Trippier et al., 2014; Jones et al., 2017; Trader et al., 2017; Santoro et al., 2020). We found that intravenous administration of pyrazolone derivative and chlorpromazine can enhance proteasome activity in pig brain.

## Materials and methods

### Animals

The animal protocol was approved by the Institutional Animal Use and Care Committee of Johns Hopkins University (protocol number SW23M119). Neonatal Yorkshire male piglets, broadly in the range of 2- to 5 days old (1–2 kg), were used for descriptive normative neuroanatomical and biochemical experiments and for drug testing experiments. Because of the different tissue needs and brain harvesting approaches for independent experiments, naïve piglets were used as separate groups for the different types of experiments. These animals were not subjected to any experimental or surgical procedures. For example, fresh brain samples were used for immunoblotting (*n* = 6 naïve piglets), optimally prepared paraformaldehyde perfusion fixed brains were used for immunohistochemistry and brightfield/confocal microscope imaging (*n* = 8 naïve piglets), and seasonal and littermate matched naïve piglets (*n* = 9) were used for the drug treatment experiments.

### Proteasome subunit analysis by immunoblotting and immunohistochemistry in pig brain at baseline

**Figure 1:**
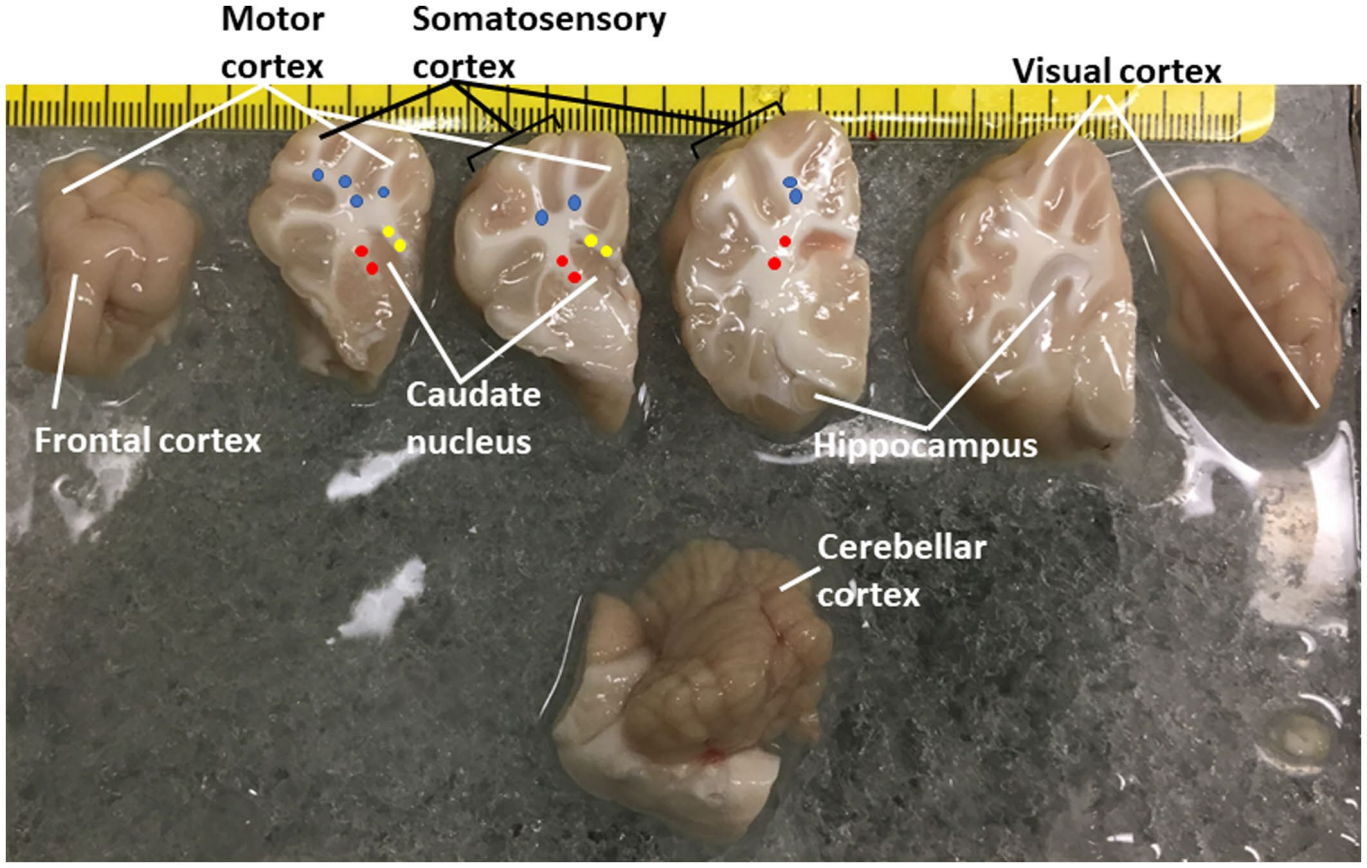
Piglet brain regions that were microdissected and used for proteasome Western blotting and biochemical or histochemical activity assays. Piglets were euthanized with Somnasol and then quickly perfused intra-aortically with ice-cold PBS for complete exsanguination. The fresh brains were quickly removed from the skull and kept ice-cold during microdissection on a chilled plate. The left hemisphere is shown in slabs from anterior (far left, frontal lobe) to posterior (far right, the occipital lobe was inadvertently rotated). The brainstem and cerebellum are shown at the bottom. The regions removed are identified. The identifications of the different areas of the neocortex are described (Primiani et al., 2023). Column micropunches of different white matter regions were taken as described (Lee et al., 2021): (blue, parietal subcortical white matter; yellow, corpus callosum; red, internal capsule). Microdissected brain samples were placed in Eppendorf tubes and snap frozen in isopentane (70°C). An additional sample of parietal somatosensory cortex was snap frozen as a small tissue block for cryostat sectioning and *in situ* proteasome activity assay.

For immunoblotting, 2- to 4-day-old naïve piglets (*n* = 6) received a lethal dose of pentobarbital 50 mg/kg and phenytoin 6.4 mg/kg (SomnaSol) and, after thoracotomy and left myocardial puncture and aortic catheterization, ice-cold 100 mM phosphate-buffered saline (PBS, pH 7.4) was perfused (~2 L) for body exsanguination. After decapitation, the brain was removed quickly and placed on wet ice. On an ice-cold metal plate, the brain was slabbed and microdissected into individual anatomical regions (Figure 1) that were snap frozen in isopentane (−70°C) cooled by a dry ice/alcohol slurry. All samples were stored in individual Eppendorf tubes at −80°C until used.

Nasal mucosa biopsy samples were also studied. The samples were obtained with a Cellebrity^™^ Cytology Brush (Boston Scientific) from sham piglets; these animals received surgical anesthesia and were instrumented for physiological monitoring. Nasal mucosa tissue was eluted from the bristles using PBS and collected by gentle centrifugation. The samples were resuspended in PBS, and contaminating red blood cells were removed using EasySep^™^ RBC Depletion Reagent (StemCell Technologies). After centrifugation, the nasal mucosa samples were frozen on dry ice and stored at −80°C until used.

Immunoblotting was done to examine the protein levels of PSMB5 and PSMA3 in different brain regions (Figure 1), their subcellular fractions, and the nasal mucosa of piglets. Frozen tissue samples were homogenized with a Brinkmann polytron in ice-cold 20 mM Tris–HCl (pH 7.4) containing 10% (wt/vol) sucrose, 200 mM mannitol, complete protease inhibitor cocktail (Roche), 0.1 mM phenylmethylsulfonyl fluoride, 10 mM benzamidine, 1 mM EDTA, and 5 mM EGTA. Crude homogenates were sonicated for 15 s and then centrifuged at 1,000 g_av_ for 10 min (4°C). For subcellular fractionation of piglet brain tissue, we used a modification of the Percoll protocol developed by Wieckowski et al. (2009). With this protocol, we isolated pure mitochondria and mitochondrial-associated membranes (MAMs) along with pure nuclear and cytosolic fractions. Protein concentrations were measured by bicinchoninic acid assay (Smith et al., 1985) with a kit (Pierce, Thermo Scientific, Carlsbad, CA) using bovine serum albumin as a standard.

Tissue lysates and subcellular fractions were subjected to sodium dodecyl sulfate polyacrylamide gel electrophoresis (SDS-PAGE) and transferred to the nitrocellulose membrane by electroelution (Martin et al., 2000; Santos et al., 2018; El Demerdash et al., 2021). Ponceau S staining of nitrocellulose membranes before immunoblotting verified the lane equivalency of sample loading and transfer in each experiment. Blots of crude tissue lysates were blocked with 2.5% non-fat dry milk with 0.1% Tween 20 in 50 mM Tris-buffered saline (pH 7.4), then incubated overnight at 4°C with rabbit polyclonal antibody to PSMB5 (GeneTex, GTX23330), a mouse monoclonal antibody to 20S proteasome (clone 863425, R&D Systems), or a rabbit monoclonal antibody to PSMA3 (clone D490, Cell Signaling Technology). To characterize the subcellular identities of the piglet brain fractions, a panel of antibodies was used to detect histone H3 (Cell Signaling Technology) for the nucleus, glyceraldehyde phosphate dehydrogenase (GAPDH) (Abcam) for the cytosol, complex V (Life Technologies) for the mitochondria, cytochrome P450 reductase (Upstate Biotechnology), and inositol triphosphate receptor (IP3R) (Fotuhi et al., 1993) for MAMs and endoplasmic reticulum. Proteasome presence in the blots of different subcellular fractions was detected using rabbit polyclonal antibody to PSMB5 (GeneTex, GTX23330) and mouse monoclonal antibody to 20S proteasome (clone 863425, R&D Systems). After the primary antibody incubation, blots were rinsed and then incubated with horseradish peroxidase-conjugated secondary antibody (0.2 μg/mL). For all blots, the primary and secondary antibodies were used at concentrations for visualizing protein immunoreactivity within the linear range. The blots were developed with enhanced chemiluminescence (Pierce) and imaged with a ChemiDoc Imaging System (Bio-Rad, Hercules, CA).

For immunohistochemistry, 2- to 5-day-old naïve piglets (*n* = 8) and sham piglets (*n* = 6) that were sedated, intubated, anesthetized, and instrumented for physiological monitoring were deeply anesthetized with pentobarbital 50 mg/kg and phenytoin 6.4 mg/kg (SomnaSol) and, after thoracotomy and left myocardial puncture and aortic catheterization, ice-cold 100 mM PBS (pH 7.4) was perfused (~2 L) for body exsanguination followed by freshly prepared 4% paraformaldehyde (PF) in 100 mM phosphate buffer (pH 7.4) for brain fixation (~4 L). Appropriate tissue fixation was judged by the stiffness of the body and the immovability of the jaw and limbs. After decapitation, the head was placed in 4% PF overnight. The following day, the calvarium was carefully removed by rongeur, and the brain was extracted from the skull base and placed in PF for overnight. Brains were cut in the coronal plane, and samples were paraffin processed in tissue cassettes. The paraffinized brain blocks were cut on a rotary microtome into 10-μm sections that were mounted on gelatin/chrome alum-coated glass microscope slides for proteasome subunit immunohistochemistry (IHC).

**Figure 2:**
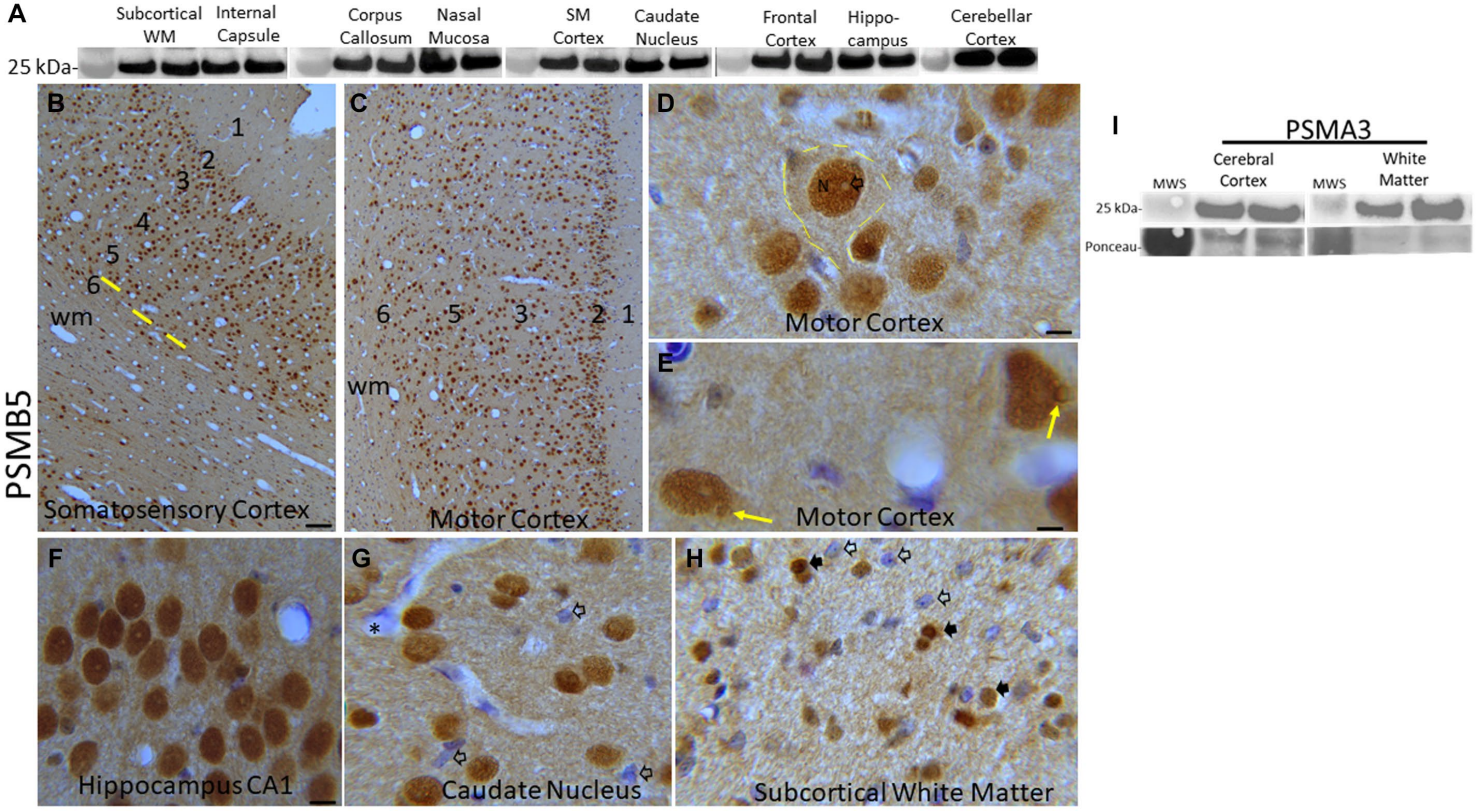
Proteasome 20S subunit PSMB5 in the piglet brain. **(A)** Western blots show that the brain regional levels of PSMB5 (detected at ~25 kDa) are relatively uniform among regions. The abundance of PSMB5 in the nasal mucosa biopsy tissue of piglets is like the different brain regions. Lanes represent regional samples from two different pigs. On the left, the 25 kDa molecular weight standard (MWS) is identified. **(B–H)** Immunohistochemical localization of PSMB5. Immunoreactivity is seen as brown staining with a blue Nissl substance (basophilic) counterstain. In primary somatosensory cortex **(B)** and motor cortex **(C)**, PSMB5 is ubiquitously present in patterns that define the horizontal neuronal layering in cerebral cortex (1–6) and vertical translaminar arrangements of neurons in somatosensory cortex (**B**, yellow lines). Essentially, all cortical neurons were PSMB5-positive **(B,C)**. A large layer 5 neuron is delineated (**D**, hatched yellow lines). PSMB5 is enriched in the nucleus (N), while the core of the nucleolus is negative (**D**, open arrow). Particulate immunoreactivity is also present in the cytoplasm and in the neuropil. **(E)** Some neurons have a discrete perinuclear inclusion (~2 μm in diameter) that is positive for PSMB5 (yellow arrows). **(F)** Essentially, all hippocampal pyramidal neurons were strongly positive for PSMB5. **(G)** Neurons in the caudate nucleus were essentially all PSMB5 positive, but capillary endothelial cells (asterisk) and subsets of glial cells (open arrows) were not positive for PSMB5. The neuropil is positive for PSMB5. **(H)** In the subcortical white matter of the parietal cortex, glial cells can be PSMB5-positive (black arrows) or -negative (open arrows). Scale bars (in μm): **(B)** (same for **C**), 40; **(D)** 7; **(E)** 4; **(F)** (same for **G,H**), 12. **(I)** Western blots show that the levels of PSMA3 (detected at ~25 kDa) are relatively uniform among regions of cerebral cortex and white matter in pig brains. Lanes represent regional samples from two different pigs.

Proteasome subunit localization IHC was done using immunoperoxidase and immunofluorescence procedures. Negative controls for both methods were anatomically matched piglet brain sections incubated with rabbit or mouse non-immunized normal IgG isotypes (Sigma-Aldrich) at the same concentrations, and for the same time, as the primary antibodies. Immunoperoxidase IHC, with diaminobenzidine (DAB) as chromogen (immunoreactivity is seen as brown), was done on piglet brain paraffin sections as described (Santos et al., 2018; Lee et al., 2021) to localize PSMB5 with rabbit polyclonal antibody (GeneTex, GTX23330). Primary antibody incubation was done overnight at room temperature in a humidified chamber. This antibody was characterized for specificity in pig brain homogenates using Western blotting. It detects a major band at ~25 kDa (Figure 2A). Nissl counterstaining with cresyl violet (CV) was done for cellular and laminar identifications in the neocortex. Immunofluorescence IHC was done on piglet forebrain paraffin sections as described (Martin et al., 2013) to identify potential subcellular colocalizations of the proteasome. PSMB5 detection was with rabbit polyclonal antibody (GeneTex, GTX23330); mitochondria were detected with mouse monoclonal antibody to cyclophilin D (clone E11AE12BD4, Abcam) and mouse monoclonal antibody to voltage-dependent anion channel (VDAC) (N152B/23, BioLegend). Cyclophilin D (also known as peptidylprolyl isomerase D, PPIF) and VDAC are well-established mitochondrial proteins, and the antibodies to these proteins have been characterized (Martin et al., 2009, 2011; Chen et al., 2021). Secondary antibodies (Thermo Fisher Scientific, Molecular Probes), used at a dilution of 1:400, were AlexaFluor-594 goat-anti-rabbit IgG and AlexaFluor-488 goat-anti-mouse IgG.

Piglet brain sections were viewed and imaged using brightfield and confocal microscopy. For brightfield microscopy, an Olympus BH-2 microscope with a CaptaVision camera and software was used. The confocal imaging was done using a Nikon C2 confocal scanning microscope equipped with a Nikon LUN4 4-line solid-state laser system, a Ti2 motorized stage, a Perfect Focus System-3, and a Nikon NIS-Elements software package or a Leica Mica Microhub Widefield Imaging System.

Confocal microscope images were used to quantify the colocalization of VDAC and PSMB5 immunoreactivities in the neuropil and neuronal perikaryal cytoplasm using a modified method described in Chang and Martin (2009). Pixel particles with green, red, and yellow variant colors were determined per 1 μm^2^ in randomly selected non-overlapping image microzones.

### PSMB5 localization in human brain

Immunofluorescence IHC was done on human cerebral cortex (postcentral gyrus and superior frontal gyrus) paraffin sections (10 μm), as described (Kageyama et al., 2018), to identify descriptively the cellular localizations of the proteasome using IHC conditions identical to those used for pig brains. Human postmortem autopsy brain samples were obtained from the Johns Hopkins Brain Resource Center as described (Martin, 1999; Kim et al., 2020). The human brain samples used here were from adult male control individuals (*n* = 3, 59–80 years old, postmortem delays to autopsy, 6–10 h) without any final diagnosis of neurologic disease and from a 1-year-old male. Staining of human and pig brain sections was done concomitantly with sodium citrate (boiling for 20 min) used for antigen retrieval and blocking/permeabilization with 10% NGS and 0.4% Triton X-100 for 2 h. PSMB5 was detected with rabbit polyclonal antibody (GeneTex, GTX23330) at 1:200. A mouse monoclonal antibody (clone 48, BD Transduction Laboratories) to the apoptosis inhibitor protein Aven was used as a general marker for the cytoplasm, nucleus, and mitochondria (Martin et al., 2007) at 1:250. Secondary antibodies were the same as those described for pigs. Coverslipping was done with VECTASHIELD^®^ containing DAPI. Piglet and human brain sections were imaged with a Mica widefield confocal scanning microscope (Leica Microsystems).

### Screening small molecules for proteasome activity activation *in vivo*

**Figure 3:**
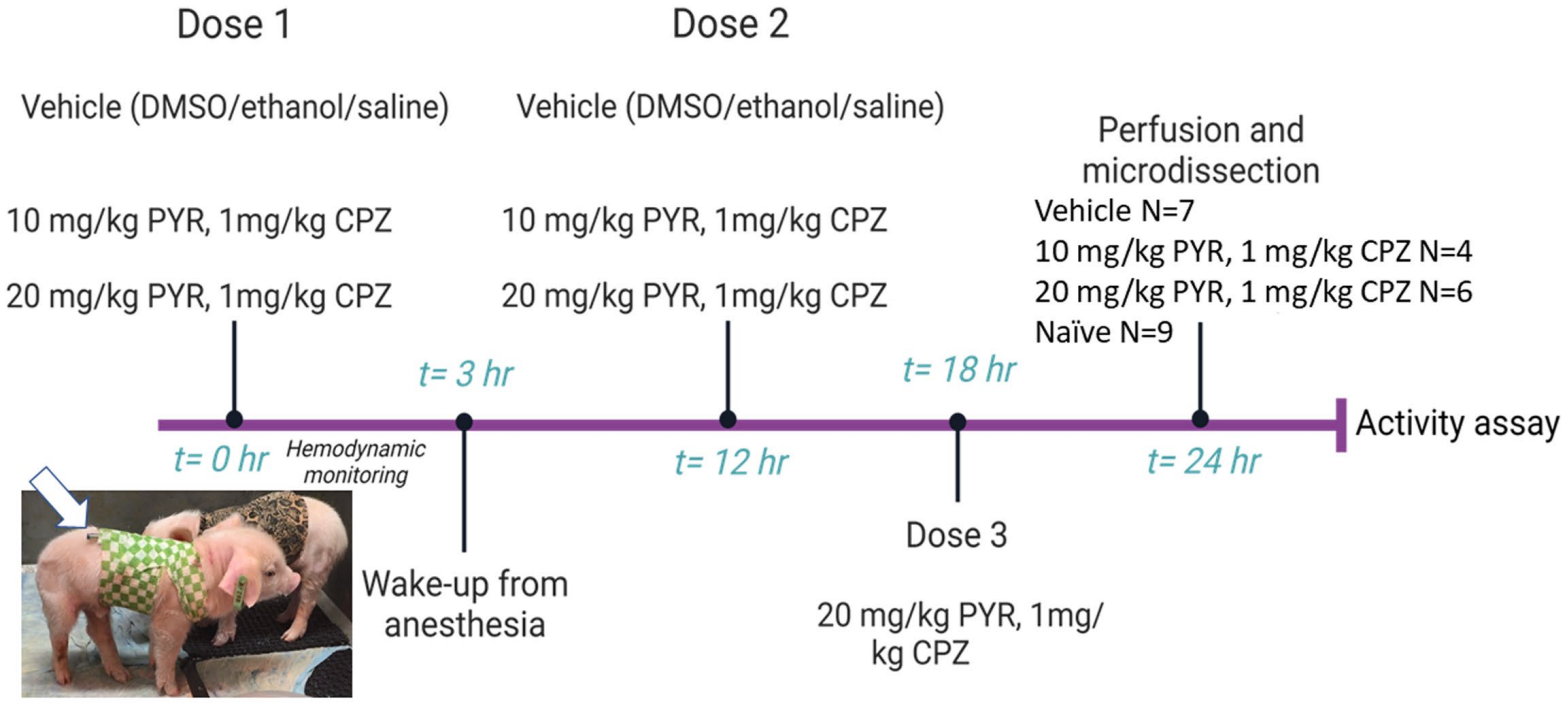
Diagram of the experimental protocol used for intravenous (iv) drug treatment of piglets. Piglets (2–3 days old, 1.5–2.0 kg, male) were sedated, intubated, and anesthetized for cardiovascular and hemodynamic monitoring before, during, and after iv administration of vehicle or a cocktail of pyrazolone (PYR) and chlorpromazine (CPZ). The piglets received multiple iv drug doses and were then euthanized for brain harvesting 24 h after the first dose (the group sizes are shown at right). Brain regional microdissections were taken (see Figure 1) and used for proteasome assays. Seasonal- and age-matched naïve piglets (*N* = 9) did not receive surgical anesthesia or any iv treatments and were euthanized with SomnaSol for immediate brain harvesting and microdissection. Animals and protocols used for other experiments (e.g., immunohistochemistry) are described specifically in the Materials and Methods.

Neonatal piglets (2–3 days old, 1.5–2.0 kg, male) were sedated, intubated, anesthetized, and instrumented with a femoral artery catheter for thorough cardiovascular monitoring before, during, and after intravenous (iv) treatment with drugs delivered through an internal jugular vein catheter tunneled to the back for central venous access to deliver additional drug doses (Figure 3). We used a narrow age range in an effort to reduce variability. Normothermic body temperature was constantly maintained with a warming blanket during the procedure. The test drugs were pyrazolone (PYR) and chlorpromazine (CPZ). The PYR derivative used was 5-[(3,5-dichlorophenoxy)methyl]-1,2-dihydro-3H-pyrazol-3-one, C_10_H_8_Cl_2_N_2_O_2_ (CMB-087229, Millipore-Sigma). The CPZ used was 2-chloro-10-(3-dimethylaminopropyl)phenothiazine hydrochloride, C_17_H_19_ClN_2_S · HCl (C8138, Sigma-Aldrich, St. Louis, MO). PYR and CPZ were identified by others (Trippier et al., 2014; Jones et al., 2017) as activators of the 20S proteasome in cell culture-based small molecule screens. After establishing hemodynamic baselines with an arterial blood pressure catheter, piglets received an iv-cocktail bolus of PYR (10 mg/kg or 20 mg/kg) and CPZ (1 mg/kg) delivered over 2 min. In independent experiments, PYR and CPZ were given alone to piglets (data now shown). PYR was well tolerated at 10 and 20 mg/kg doses. However, higher concentrations of CPZ (>10 mg/kg) produced unacceptable lethargy, hypotonia, and acquired dystonia in piglets. This is why a lower dose of CPZ was used. Control piglets received vehicle (iv DMSO/ethanol/saline). About 3 h later, after blood pressure and heart rate monitoring, the piglets had their arterial catheter removed and the wound closed. They were awakened from anesthesia and extubated. A second dose of PYR/CPZ or vehicle was delivered iv 12 h after the first dose. A subset of piglets received a third iv dose of PYR/CPZ or vehicle 6 h later (because of insufficient power, these animals were combined into the total group of piglets receiving 20 mg/kg PYR and 1 mg/kg CPZ).

Twenty-four hours after the initial dosing, the piglets received an overdose of SomnaSol and were quickly thoracotomized for cardiac puncture, aortic cannulation, and exsanguinated with ice-cold PBS perfusion (~2 L). Effective and complete exsanguination was necessary for removing proteasome-rich erythrocytes, with their ~20-fold excess of 20S proteasome over 26S (Neelam et al., 2011), from the brain. Brain gray and white matter regions (Figure 1) were harvested for proteasome chymotrypsin-like (CTL) activity (Brown and Monaco, 1993; Kisselev and Goldberg, 2005), Western blotting, an in-gel proteasome activity assay (Yazgili et al., 2021), or a novel *in situ* proteasome assay.

Native protein extracts were used for biochemical assays of functional proteasomes. Tissue samples were homogenized and sonicated in ice-cold tissue lysis buffer (40 mM Tris and 50 mM NaCl in distilled water at pH 7.2 with 10% glycerol), 2 mM β-mercaptoethanol (Sigma-Aldrich), 5 mM MgCl_2_, and 2 mM adenosine 5′-triphosphate (ATP) (UBPBio, Dallas, TX). The samples were then centrifuged for 30 min at 4°C. The supernatant was collected and kept on ice for protein measurements using a bicinchoninic acid assay kit (Smith et al., 1985; Pierce). Proteasome CTL activity was measured using a fluorometric assay kit (UBPBio). Proteasome assay buffer was prepared by adding 50 μL of 20× proteasome assay buffer to 950 μL of sterile water at 37°C. The substrate was prepared by adding 2 μL of 1,000× Succinyl-Leu-Leu-Val-Tyr-7-amino-4-methylcoumarin (Suc-LLVY-AMC) stock to the assay buffer. All samples (50 μg total protein input) were assayed in duplicate with and without MG132 in 96-well, black-bottom plates. The proteasome inhibitor MG132 (Lee and Goldberg, 1998) was added to a parallel set of sample wells to distinguish peptidase activities. Suc-LLVY-AMC is also degraded by calpains, cathepsins, and other CTL proteases (Rock et al., 1994). Adding MG132 isolates proteasome substrate cleavage from non-proteasome proteolysis. Each plate included an AMC standard (blank, 1:500, 1:1,000, 1:5,000, and 1:10,000) to generate a concentration-dependent AMC fluorescence standard curve. Liberated AMC fluorescence from proteolysis of Suc-LLVY-AMC was immediately measured by a plate reader (TECAN Infinite M Plex) with 360 nm excitation/460 nm emission filters for 15 min of assay reaction time at 37°C. Each sample’s AMC fluorescence was determined from the AMC standard curve and reported as relative fluorescence units. The AMC fluorescence from tissue homogenate with MG132 was subtracted from that of homogenate without MG132 to obtain relative fluorescence levels specific to proteasome Suc-LLVY-AMC peptidolysis. Replicates were averaged, and their corresponding MG132-controlled wells were subtracted. Femtomoles of AMC released were calculated from relative fluorescence units using a standard curve of purified AMC included in each microplate assay. The AMC standard curve slope for each plate was standardized to the average activity slope of MG132-inhibited wells with cerebellar cortex input.

For the in-gel proteasome activity assay, we used the same tissue homogenates that were prepared under non-denaturing conditions and used for the proteasome fluorometric microplate assay. Native PAGE was done using 3–8% Tris-acetate gels. Purified human 20S from human erythrocytes (Enzo Life Sciences) was used as a positive control. The resulting gels were incubated for 30 min at 37°C in a dark box in a reaction solution containing Tris (100 mM, pH 7.5), ATP/MgCl_2_, dithiothreitol (DTT), and Suc-LLVY-AMC (2 mM). Negative control gels were incubated in a reaction solution containing MG132 (10 μM). Proteasome CTL enzymatic activity in gels was imaged with a ChemiDoc imaging system (Bio-Rad).

We developed a novel *in situ* proteasome enzyme histochemical method to localize active proteasome in brain sections. Freshly frozen (unfixed) blocks of piglet somatosensory were cut (10 μm thick) on a cryostat and mounted on gelatin/chrome alum-coated glass microscope slides. The slides were stored at −80°C in closed boxes. Sections were incubated in the dark for 2 h at 37°C with a CTL proteasome reaction solution containing 100 mM Tris (pH 7.5), 250 mM ATP/MgCl_2_, 1 mM DTT, 50 mM Suc-LLVY-2R110 (AAT Bioquest), and 20% polyvinyl alcohol. Negative control sections were pretreated with MG132 and incubated in a reaction solution containing MG132 (10 μM). Afterwards, the slides were rinsed in Tris buffer, dipped in 4% PF/20% sucrose for 5 min, counterstained with DAPI, and coverslipped. Sections were viewed with a Zeiss Axiophot microscope under epifluorescence and imaged with SPOT software. Suc-LLVY-2R110, upon CTL cleavage, generates a fluorescent product that is bright green (excitation 498 nm/emission 520 nm) when viewed with a fluorescein isothiocyanate filter set.

### Statistical analysis

GraphPad Prism 9 was used to analyze data. Brain regional proteasome activity data passed Shapiro–Wilk tests for normality and were compared using Kruskal–Wallis ANOVA on ranks. Correlations between nasal biopsy and brain regional proteasome activities were examined by Spearman correlation.

## Results

### Proteasome protein levels and localization in neonatal pig brain

Western blotting was done to determine relative protein levels of the β5 proteasome subunit (PSMB5) in different regions of the neonatal pig brain (Figure 2A). Immunoreactivity for the β5 proteasome subunit was detected at ~25 kDa, consistent with expectations from the work of others (Sutovsky et al., 2004) and us (Santos et al., 2018; El Demerdash et al., 2021). In precisely microdissected white matter regions (Figure 1) such as parietal cortex subcortical white matter, corpus callosum, and internal capsule β5 levels were similar (Figure 2A). Microdissected gray matter regions of cerebral cortex (primary somatosensory cortex and frontal cortex) hippocampus, caudate nucleus, and cerebellar cortex β5 levels were similar to each other and to white matter (Figure 2A). Nasal mucosa biopsy samples from piglets also had enrichment of β5 immunoreactivity like the different brain regions (Figure 2A). From the Western blotting, we deemed the PSMB5 antibody suitable for immunohistochemical application on pig brain (Figures 2B–H). The α3 proteasome subunit (PSMA3) was also examined in pig brain by Western blotting (Figure 2I). The PSMA3 immunoreactive band was also detected at ~25 kDa, like the mobility of PSMB5 (Figure 2A). In selected brain regions, PSMA3 levels were similar in gray matter and white matter areas (Figure 2I).

IHC was used to localize PSMB5 in different regions of the piglet brain (Figures 2B–H and Supplementary Figures 1A–C). PSMB5 immunoreactivity was ubiquitous and not particularly defining of any brain regions. Essentially, every neuron in each layer of the cerebral cortex was positive (Figures 2B–E). The somatosensory cortex (Figure 2B) had a distinct layer 4 that was inconspicuous in the motor cortex (Figure 2C), consistent with other observations (Primiani et al., 2023). In addition to the horizontal laminar patterns, vertical column arrangements of cell bodies, organized orthogonally to the cortical surface, were visible in the primary somatosensory cortex (Figure 2B, yellow lines). These cortical columns had a width of 4–5 neuronal perikarya (40–50 μm in total) and a periodicity of about 50 μm. Virtually all pyramidal neurons in the hippocampus were positive for PSMB5 (Figure 2F). The principal neurons in the striatum were generally positive (Figure 2G). Glial cells in gray matter and white matter were positive or negative for PSMB5 (Figures 2E,G,H). PSMB5 was localized conspicuously in the nuclei of neurons (Figures 2D–G) and glia (Figures 2D,G,H) where it was strongly in the nucleoplasmic matrix but typically not within the nucleolus. In the cytoplasm and neuropil, PSMB5 immunoreactivity had an amorphous diffuse and a particulate appearance (Figures 2D,G).

### Proteasome protein localization in pig and human neocortex

We performed an experiment to compare the localization of PSMB5 in neonatal pig neocortex to young and adult human disease-free neocortex concomitantly using identical immunofluorescence methods (Supplementary Figure 1). In pigs (Supplementary Figures 1A–C), the PSMB5 staining pattern seen by immunofluorescence was like that seen by immunoperoxidase (Figures 2B–E). There was prominent nuclear localization in virtually all neurons in each cortical layer. PSMB5 was also present in the cytoplasm as a diffuse overlapping pattern with immunoreactivity for the antiapoptotic protein Aven (Supplementary Figures 1B,C).

The patterns of PSMB5 immunoreactivity in human neocortex in young (1-year-old) and aged (59–80 years old) individuals (Supplementary Figures 1D–H) were variations of the pattern seen in neonatal pigs. In the 1-year-old brain, most neocortical neurons in all layers were PSMB5-positive with prominent nuclear labeling but low colocalization with Aven (Supplementary Figures 1D–F). There was an inhomogeneity of immunoreactivity in the neuropil, with some areas slightly higher in PSMB5 immunoreactivity than nearby areas (Supplementary Figure 1D, white hatched outlines). In the older human brain, neurons were positive or negative for PSMB5 (Supplementary Figures 1G,H) compared to virtually all cerebrocortical neurons being positive in neonatal pig and young human brain (Figures 2B–E and Supplementary Figures 1A–C). The superficial layers (2 and 3) had more prominent neuropil PSMB5 immunoreactivity than the deeper layers. Positive neurons in aged human brains generally had more apparent PSMB5 immunoreactivity in the cytoplasm than in the nucleus (Supplementary Figure 1H), while in neonatal pigs and young human brain, PSMB5 nuclear immunoreactivity was prominent. The Aven immunoreactivity in the cytoplasm was very discreetly and strongly colocalized with PSMB5 in older human neocortical neurons in a pattern suggestive of mitochondria (Supplementary Figures 1E,F), which contrasted with the infant brain.

### Detection of mitochondrial-associated proteasome CTL activity

We performed a bioinformatic analysis on the human PSMB5 full-length amino acid sequence using MitoFates (Fukasawa et al., 2015) to search for mitochondrial targeting and mitochondrial processing peptidase (MPP) cleavage sites. This software has better discriminative and predictive performance than other predictive tools (Fukasawa et al., 2015). A well-known mitochondrial matrix protein, CyPD (PPIF), was used as a comparator protein. PSMB5 registered a low probability N-terminal mitochondrial targeting sequence (probability = 0.002) compared to CyPD (probability = 0.982). However, PSMB5 had an N-terminal MPP site in the vicinity of amino acid 17 and a C-terminal VKKVI TOM20 recognition motif (Saitoh et al., 2007).

**Figure 4:**
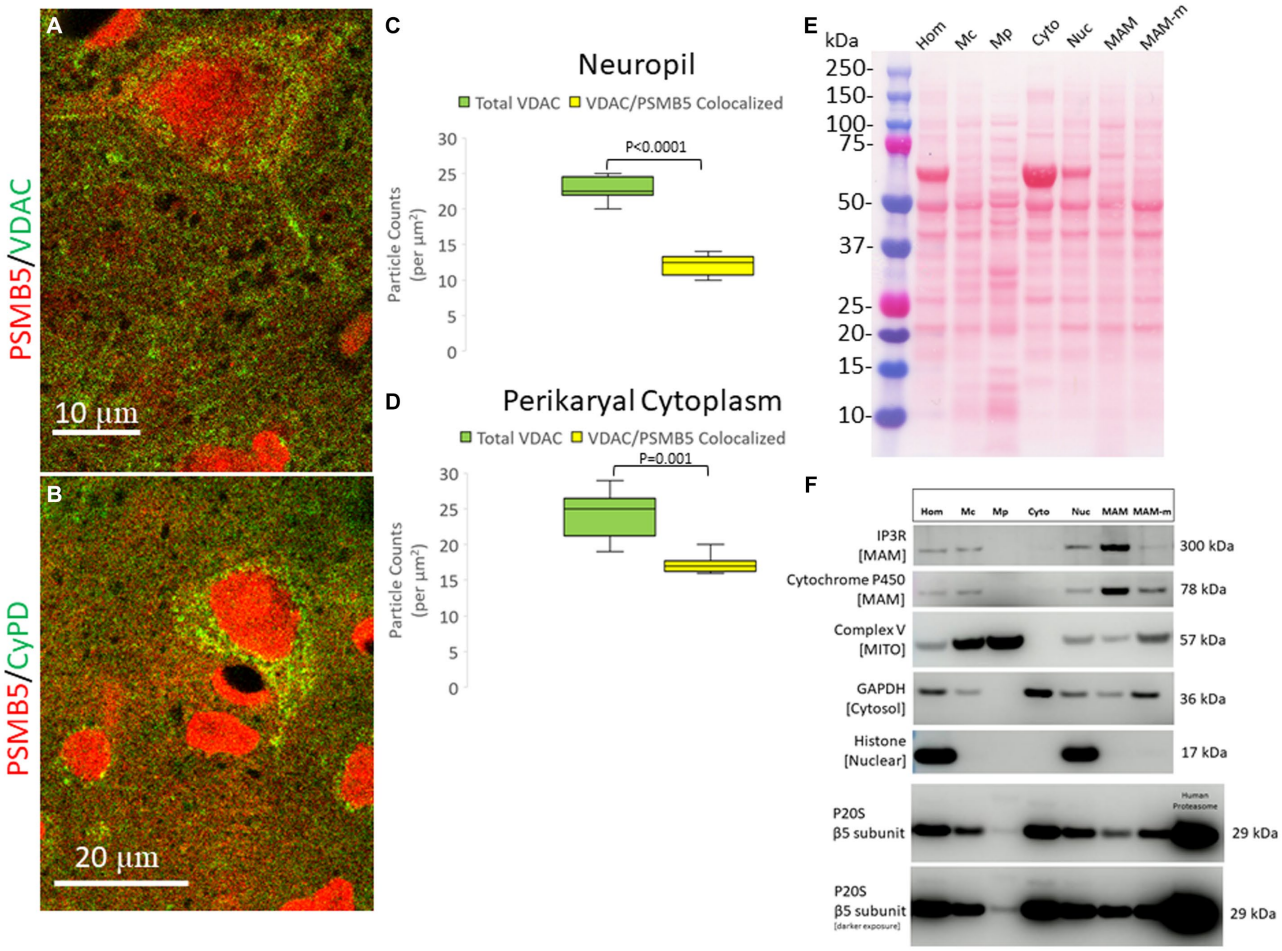
Subcellular localization of the proteasome seen by confocal microscopy and brain tissue fractionation. **(A)** Confocal microscope image shows PSMB5 (red) and voltage-dependent anion channel (VDAC, green) as a mitochondrial marker. Some mitochondria are green only, and some are yellow (corresponding to mitochondria-proteasome colocalization). Other mitochondria are seen with proteasome immunoreactivity associated with the outer surface (a discrete green profile with closely associated red particles on the surface). Colocalization of proteasomes with mitochondria (yellow) is seen within the neuropil, perhaps corresponding to synaptic mitochondria proteasomes. **(B)** Confocal microscope image shows PSMB5 (red) and cyclophilin D (CyPD, green) as a mitochondrial marker. **(C)** Colocalization graph for VDAC-positive mitochondria located in the neuropil and PSMB5. Box plots show mean values with IQR and 5–95th percentile whiskers. **(D)** Colocalization graph for VDAC-positive mitochondria located in the neuronal perikaryal cytoplasm and PSMB5. Box plots show mean values with IQR and 5–95th percentile whiskers. **(E)** A representative nitrocellulose membrane with transferred proteins after SDS-PAGE and Ponceau S staining shows the exquisitely resolved protein banding patterns in different subcellular fractions resulting from Percoll ultracentrifugation of piglet somatosensory cortex homogenates. Somatosensory cortex was homogenized (Hom) and then fractionated into the following subcellular compartments: nuclear (Nuc), cytosolic (Cyto), crude mitochondria (Mc), pure mitochondria (Mp), mitochondrial-associated membranes-mitochondrial pellet (MAM-m), and pure mitochondrial-associated membranes (MAM). At left, the molecular weight kDa standards (Precision Plus Protein Dual Color Standards, Bio-Rad) are identified. **(F)** Western botting was done to characterize the fractions with antibodies to inositol triphosphate receptor (IP3R), cytochrome P450 reductase, mitochondrial complex V (ATP synthase), glyceraldehyde phosphate dehydrogenase (GAPDH), and histone H3. PSMB5 was detected in all fractions, including the pure mitochondrial fraction. Purified human P20S was a positive control.

The subcellular localization of PSMB5 was explored directly in optimally prepared pig brains using immunofluorescence and confocal microscopy. PSMB5 immunoreactivity was enriched in the nucleus and cytoplasm (Figures 4A,B and Supplementary Figures 1A–C). PSMB5 colocalized partially with two different well-known mitochondrial markers (VDAC and cyclophilin D) in the cytoplasm of neurons and in the neuropil of the cerebral cortex and striatum (Figures 4A,B). Quantification of the colocalization showed that about half of the total mitochondria in the neuropil and perikaryal cytoplasm detected with VDAC colocalized with PSMB5 (Figures 4B,C). There was a significantly greater presence of PSMB5 in perikaryal cytoplasmic mitochondria compared to neuropil mitochondria (Figures 4B,D).

To confirm that the proteasome is possibly associated with mitochondria, piglets were completely exsanguinated by vascular perfusion of ice-cold PBS (to remove proteasome-enriched contaminant erythrocytes), and the fresh (not frozen) brain tissue was used immediately for subcellular fractionation into nuclear, cytosolic, crude mitochondrial, pure mitochondria, and mitochondrial-associated membranes (Figure 4E). The effectiveness of the subcellular fractionation method was confirmed using a panel of antibodies to different cellular constituents (Figure 4F). Histone H3, seen as a 17 kDa band, was detected only in the crude homogenate and in the nuclear fraction (Figure 4F). Complex V (ATP synthase), seen as a 57 kDa band, was concentrated in the pure mitochondrial fraction (Figure 4F). Critically, GAPDH, a cytosolic marker, was not present in the pure mitochondria fraction (Figure 4F), and thus, the pure mitochondrial fraction was deemed free of cytosolic proteasome contamination. The MAM fraction was enriched in IP3R, seen at 260–300 kDa, and cytochrome P450 reductase, detected at ~78 kDa (Figure 4F).

### Subcellular apportioning of proteasome CLT activity in neonatal pig brain

**Figure 5:**
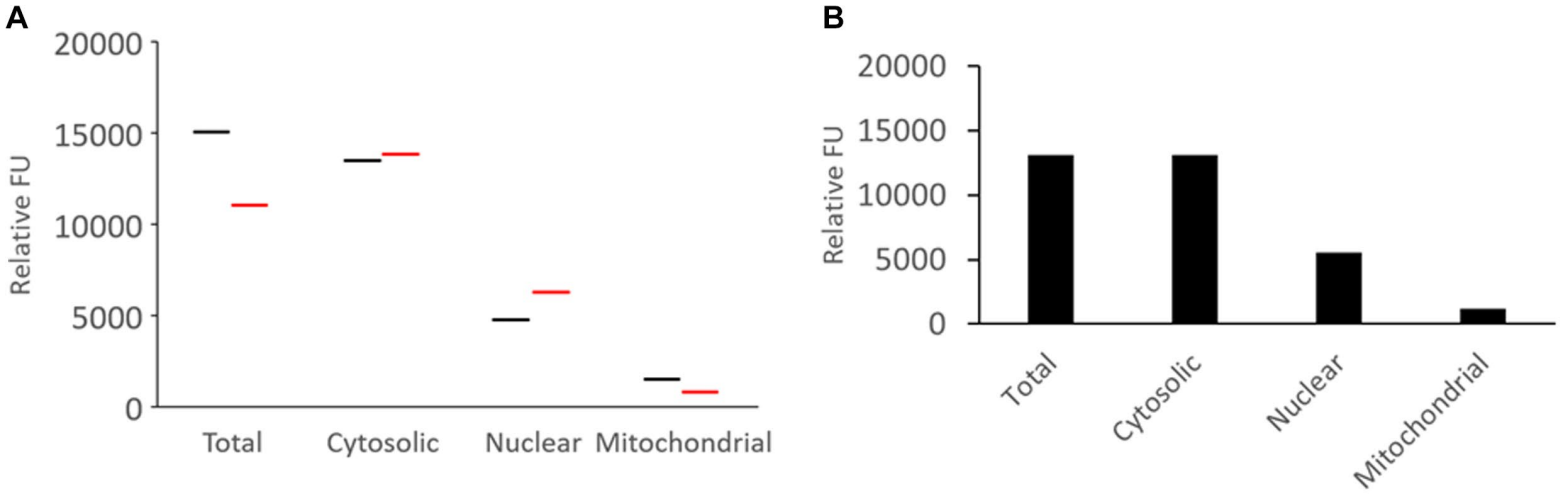
Proteasome CTL activity in subcellular fractions of the somatosensory cortex of naïve piglets. **(A)** Brain tissue from two different piglets was subjected to subcellular fractionation and assayed for proteasome CTL activity in the different fractions. The UBPBio fluorogenic assay was done with Suc-LLVY-AMC as the substrate. Each line (black or red) represents a single piglet brain sample. **(B)** Mean of the proteasome CTL activities in the different subcellular fractions (*n* = 2 different pigs).

Fresh unfrozen naïve piglet brain (*N* = 2) was microdissected and subjected to tissue subcellular fractionation into cytoplasmic (soluble), nuclear, and pure mitochondrial compartments that were compared to the total activity in crude sonicated homogenate (Figure 5). Western blotting confirmed the brain tissue subcellular fractionation method (Figure 4F). The results were remarkably consistent among different pigs (Figure 5A), though this is only a descriptive pilot experiment with a small number of naïve piglet brains. The majority of proteasome CTL activity was in the cytosolic fraction (Figure 5B). About 40% of the total activity was in the nucleus (Figure 5B), and about 15–20% was in the pure mitochondrial fraction (Figure 5B).

**Figure 6:**
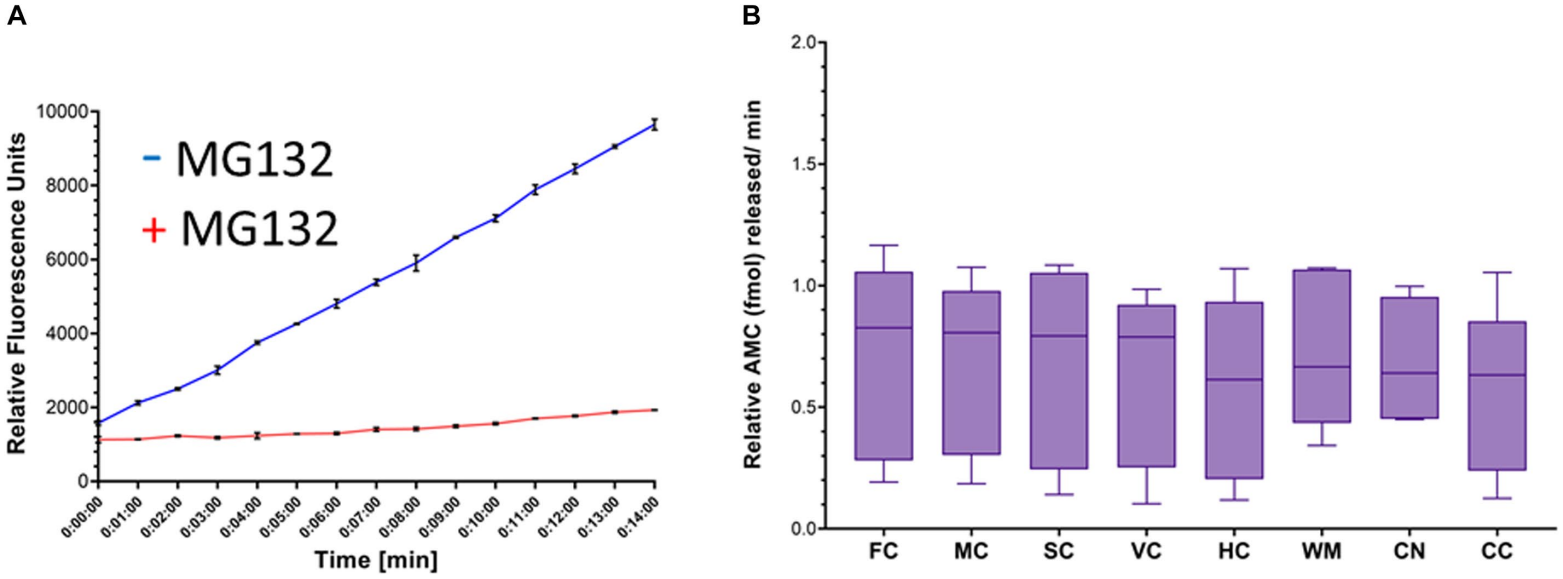
Proteasome CTL activity in crude homogenates of different brain regions of naïve piglets. **(A)** Proteasome fluorogenic assay shows reaction progress curve raw fluorescence measurements of AMC released from Suc-LLVY-AMC by chymotrypsin-like activity over 14 min in the somatosensory cortex of a single naïve piglet. To isolate the specific fluorescent signal produced by AMC liberation due to proteasome activity, MG132 was used as an inhibitor in separate duplicate control wells. **(B)** Boxplots of femtomoles (fmol) AMC released per minute by proteasome CTL activity in each brain region in naïve piglets (*N* = 9). The individual brain regions are: FC, frontal cortex; M, motor cortex; SC, somatosensory cortex; VC, visual cortex; HC, hippocampus; WM, subcortical white matter; CN, caudate nucleus; CC = cerebellar cortex. Each box plot shows the minimum/Q1/median/Q3/maximum values.

The proteasome CTL activity assay was validated. Proteasome activity was detected as an increase in fluorescence units over time that was blocked effectively by MG132 (Figure 6A). Total AMC fluorescence at the reaction termination (15 min) was reduced by ~85% by MG132 (Figure 6A). This level of proteasome inhibition was reproduced in every independent assay (more than 20 different experiments) and was shown elsewhere (El Demerdash et al., 2021).

### Proteasome biochemical activity in different brain regions of naïve piglets

**Figure 7:**
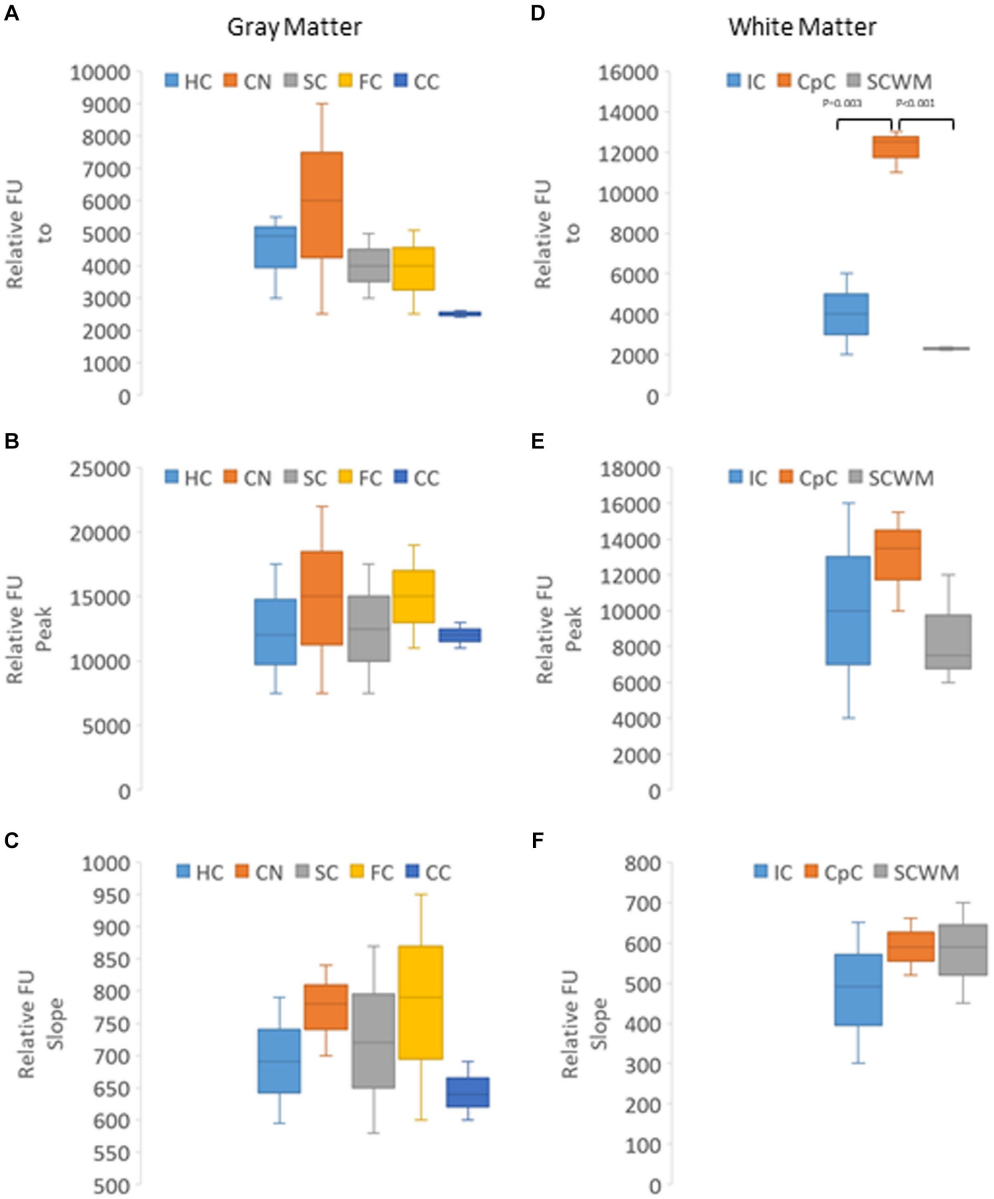
Kinetic analysis of proteasome CTL activity in different gray and white regions of the brain of naïve piglets (*N* = 4 or 5 for each brain region). **(A–C)** Relative AMC fluorescence at initial reaction time identified as t0 **(A)**, peak fluorescence **(B)**, and slope of the progress reaction curve **(C)** in hippocampus (HC), caudate nucleus (CN), primary somatosensory cortex (SC), frontal cortex (FC), and cerebellar cortex (CC). **(D–F)** Relative AMC fluorescence at *t*_0_ **(D)**, peak fluorescence **(E)**, and slope of the progress reaction curve **(F)** in the internal capsule (IC), corpus callosum (CpC), and parietal subcortical white matter (SCWM). Box plots show mean values with IQR and 5–95th percentile whiskers.

**Figure 8:**
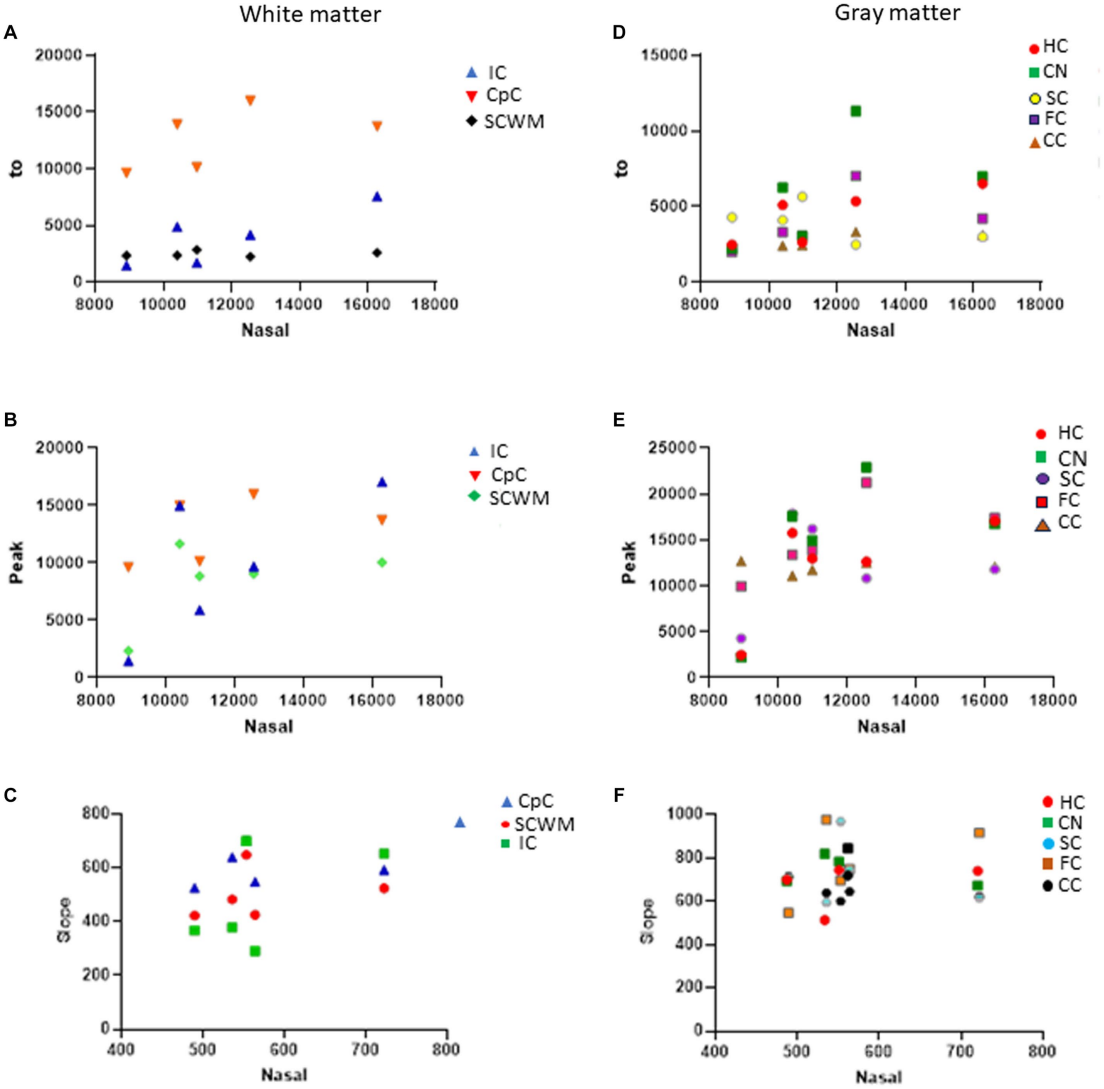
Proteasome CTL activity reaction progress curve properties (fluorescence *t*_0_, peak, slope) in different white and gray regions of sham piglet brains (*N* = 4 or 5) versus proteasome activity in nasal mucosa biopsy samples of the same piglets. **(A–C)** Scatterplots show the white matter regions of the internal capsule (IC), corpus callosum (CpC), and parietal subcortical white matter (SCWM). **(D–F)** Scatterplots show the gray matter regions of the hippocampus (HC), caudate nucleus (CN), primary somatosensory cortex (SC), frontal cortex (FC), and cerebellar cortex (CC).

In naïve neonatal pigs, no significant difference in activity was observed among different brain regions, including gray and white matter areas (Figure 6B). Proteasome activity in different gray and white matter regions was examined based on initial *t*_0_ activity, peak fluorescence (the end of the reaction progress curve), and the slope of the reaction progress curve. These parameters did not differ among brain regions, except for the corpus callosum (Figure 7). The *t*_0_ proteasome activity in the corpus callosum was significantly higher (*p* < 0.05) compared to the internal capsule and the parietal subcortical white matter (Figure 7D). Proteasome activities in the nasal mucosa and in different brain regions were examined for correlations (Figure 8). In white matter, no significant correlations were observed (Figures 8A,B). For example, there was no relationship between proteasome peak activity in the internal capsule and nasal mucosa proteasome activity (Spearman’s *r* = 0.80, *p* = 0.14). However, the proteasome activities in the nasal mucosa and some gray matter regions were correlated (Figures 8D–F). Proteasome *t*_0_ activity in the hippocampus is correlated with nasal mucosa proteasome activity (Spearman’s *r* = 0.9746, *p* = 0.0001). Proteasome peak activity in the hippocampus is correlated with nasal mucosa proteasome activity (Spearman’s *r* = 0.900, *p* = 0.02). In contrast, the hippocampal proteasome activity slope did not correlate with nasal mucosa proteasome activity (Spearman’s *r* = 0.632, *p* = 0.23). Caudate nucleus proteasome *t*_0_ activity did not correlate with nasal mucosa proteasome activity (Spearman’s *r* = 0.80, *p* = 0.065).

### Proteasome biochemical activity in different brain regions of PYR/CPZ-treated piglets

Piglet hemodynamics were monitored for 3 h after iv drug/vehicle delivery, after which piglets were recovered from anesthesia and survived. The average mean arterial pressure was 65 ± 4 mmHg in drug-treated piglets and 72 ± 6 mmHg in vehicle-treated piglets. The average heart rate was 166 ± 18 beats/min in drug-treated piglets and 198 ± 25 beats/min in vehicle-treated piglets.

**Figure 9:**
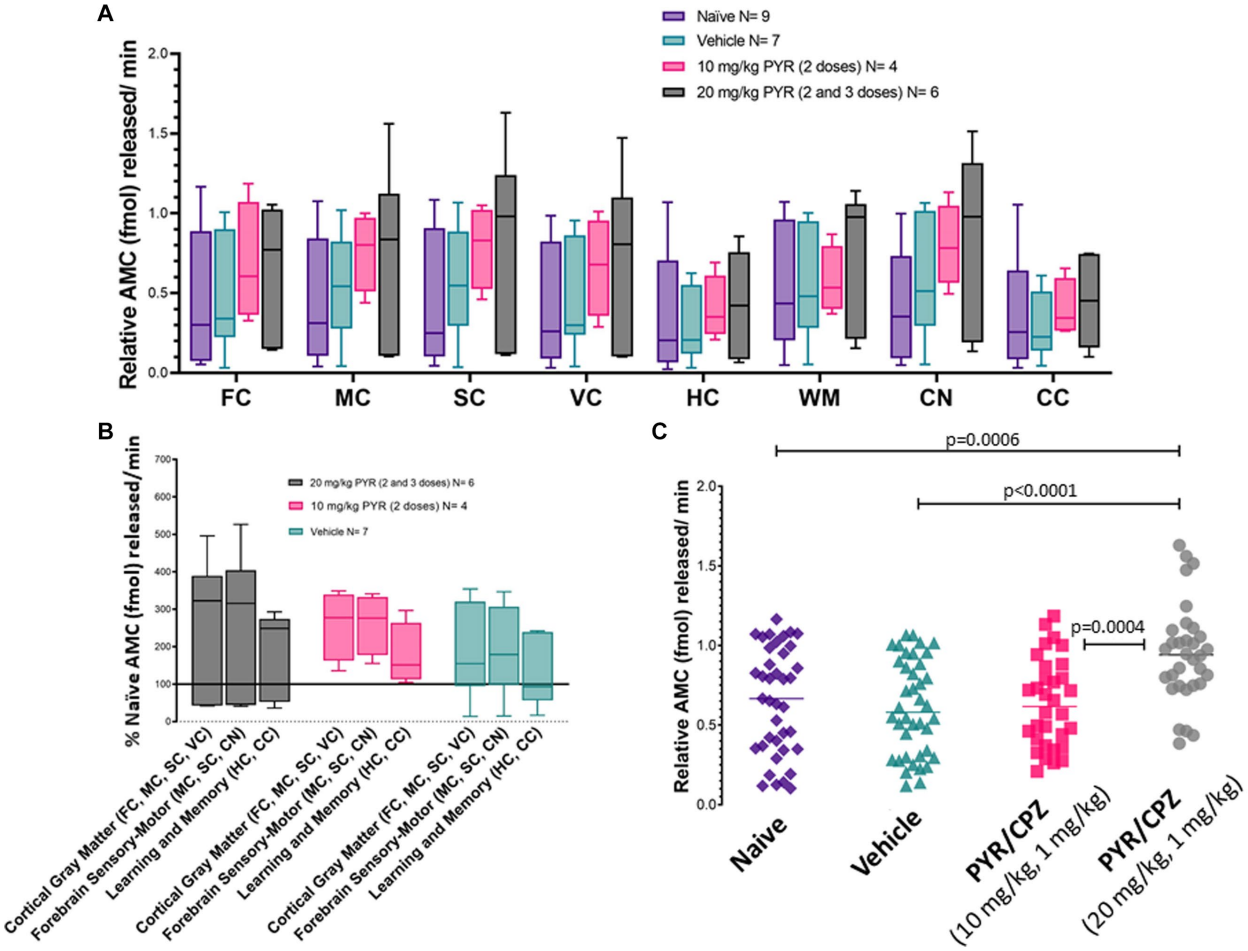
Brain proteasome CTL activity in drug-treated piglets. **(A)** Box plots of femtomoles (fmol) of AMC released per minute by proteasome chymotrypsin-like activity in each brain region in the different piglet treatment groups identified. Each box plot shows the minimum/Q1/median/Q3/maximum values. The individual brain regions are: FC, frontal cortex; M, motor cortex; SC, somatosensory cortex; VC, visual cortex; HC, hippocampus; WM, parietal subcortical white matter; CN, caudate nucleus; CC, cerebellar cortex. Each box plot shows the minimum/Q1/median/Q3/maximum values. **(B)** Proteasome activity in brain regions grouped by functional relationships. Each box plot shows the minimum/Q1/median/Q3/maximum values. **(C)** Combined brain region holistic scatterplots of fmol AMC released per minute by proteasome chymotrypsin-like activity in each treatment group. Lines are medians. The significance was determined by two-way ANOVA (*α* = 0.05) of multiple comparisons between treatment groups.

Piglets were treated (2–3 doses) with iv 10 mg/kg PYR or 20 mg/kg PYR with both dosages in a cocktail with 1 mg/kg CPZ (Figure 3). Twenty-four hours after the first dose, brain tissues (Figure 1) were harvested freshly for proteasome assays (Figure 9). In an analysis of eight different brain regions, though trends were apparent for dose-related changes in proteasome activity with PYR/CPZ, proteasome activity was not increased significantly (*p* < 0.05) above vehicle or naïve for any brain region (Figure 9A). When brain regions were stratified by functional network, trends were again apparent for increased proteasome activity with PYR/CPZ, but they did not achieve significance (Figure 9B). When all brain regions were grouped (Figure 9C), piglets treated with 20 mg/kg PYR/1 mg/kg CPZ had significantly higher proteasome activity compared to naïve piglets (*p* = 0.0006), vehicle-treated piglets (*p* < 0.0001), and 10 mg/kg PYR/1 mg/kg CPZ-treated piglets (*p* = 0.0004). Piglets treated with 10 mg/kg PYR/1 mg/kg CPZ did not differ significantly from naïve and vehicle-treated piglets.

Western blotting was done to determine if PYR/CPZ treatment altered the levels of PSMB5 in the somatosensory cortex (Supplementary Figure 2A) at 24 h after treatment (see Figure 3 for treatment protocol). PSMB5 levels were not significantly different (*p* > 0.05) in drug-treated piglets compared to vehicle-treated piglets (Supplementary Figure 2B).

### In-gel proteasome biochemical activity in pig cerebral cortex

**Figure 10:**
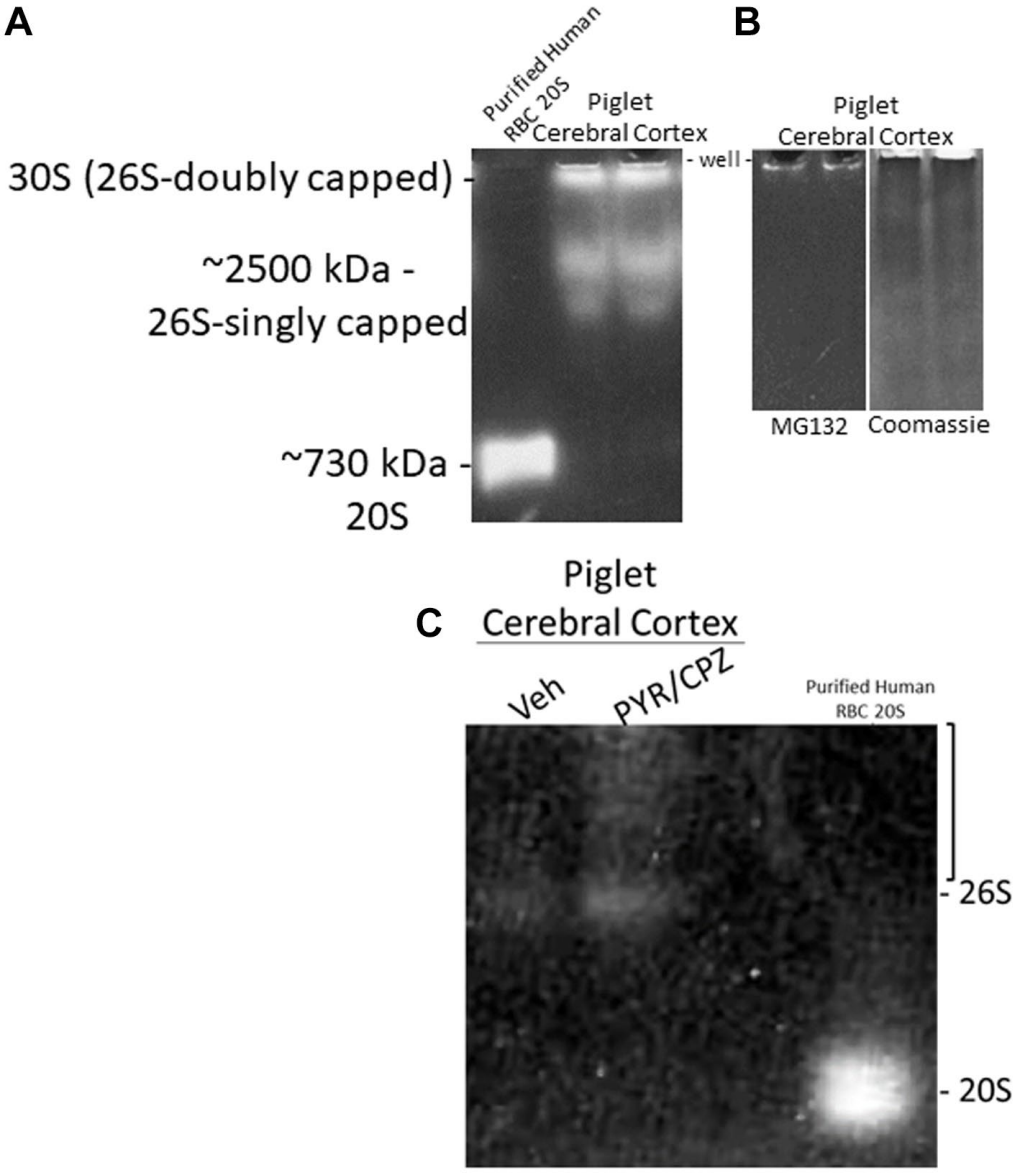
In-gel proteasome CTL activity in piglet cerebral cortex. **(A)** Protease activity gel shows the presence of the 26S (singly capped) and 30S (26S-doubly capped) proteasomes in the piglet motor cortex. Purified proteasome 20S from human erythrocytes was used as a standard. **(B)** 26S and 30S proteasome activities in piglet cerebral cortex were inhibited by the standard proteasome inhibitor MG132 (left gel). Representative gel loading verification by Coomassie staining (right gel). **(C)** In-gel proteasome CTL in piglet cerebral cortex showed enhanced activity with PYR/CPZ treatment compared to vehicle. Enhancement of activity occurred with the 26S proteasome and as a smear of activity (bracket) above the 26S-singly capped.

We did in-gel assays to determine the activity and composition of functional proteasome complexes in the piglet cerebral cortex (Figure 10). In-gel proteasome assays revealed 26S-singly capped and 30S (26S-doubly capped) active complexes in piglet neocortex (Figure 10A). These activity bands were inhibited by 10 μM MG132 (Figure 10B), demonstrating that they are proteasomes (Lee and Goldberg, 1998). We did not identify free 20S proteasome in the piglet motor cortex (Figure 10A).

In a side-by-side descriptive comparison of in-gel activities with short incubation times, piglets treated with PYR/CPZ showed enhanced neocortical proteasome activity compared to vehicle-treated piglets. Activity enhancement in somatosensory cortex with PYR/CPZ treatment occurred in the 26S-singly capped proteasome and as a smear of activity between 26S-singly capped and 30S (Figure 10C). No enhancement of activity was seen for free 20S.

### *In situ* proteasome histochemical activity in piglet cerebral cortex

**Figure 11:**
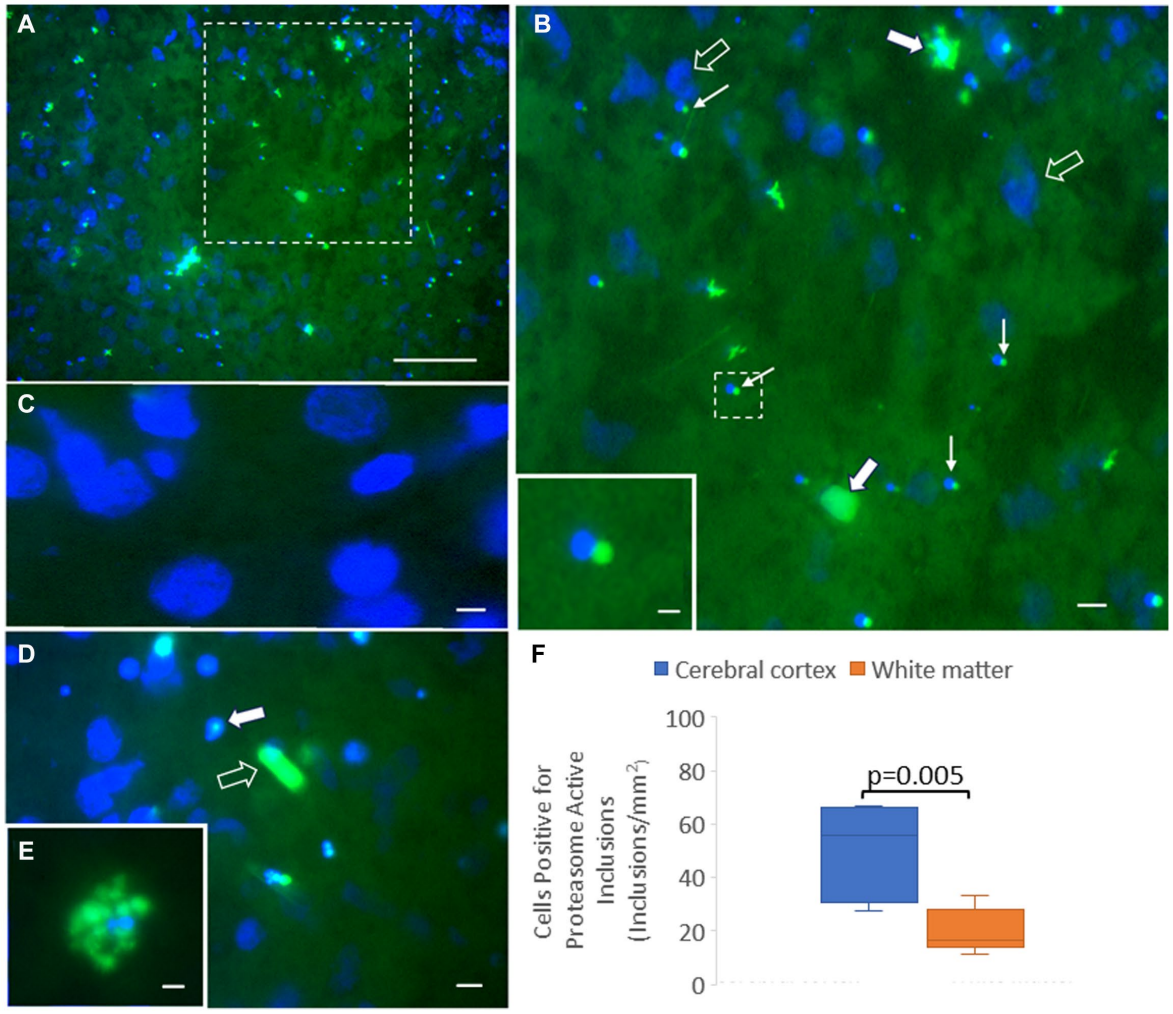
Novel *in situ* histochemical detection of proteasome activity in piglet brain cryostat sections reveals new findings on proteasome localization. **(A)** Proteasome CTL activity (green) is enriched (but not uniformly) in the neocortical neuropil and in subsets of cells in the somatosensory cortex of piglet. Blue is the DAPI counterstaining of the nucleus. The white hatched box is shown at higher magnification in panel **(B)**. **(B)** Large cells (solid white arrows), likely neurons, are enriched in proteasome CTL activity, but other neurons (open arrows; blue is DAPI staining of the large nucleus) appear without detectable activity. Some small cells (thin white arrows) have perinuclear inclusions with high proteasome CTL activity. The white hatched box is shown as the inset to better illustrate the proteasome-active perinuclear satellite. **(C)** MG132 treatment of sections completely inhibited the histochemical detection of proteasome activity (specific green reaction product) in the neuropil and in cell bodies (blue DAPI staining shows cell nuclei). **(D)** Some cells in the neocortex have proteasome activity focally within the nucleus (solid white arrow) with limited to no activity in the cytoplasm, while other cells appear completely enriched in proteasome activity (open white arrow). **(E)** A cell in the cerebral cortex highly enriched in proteasome activity. **(F)** Graph of the number of cells in cortical gray matter and subcortical white matter with perinuclear proteasome-active inclusions. Box plots show mean values with IQR and 5–95th percentile whiskers. Scale bars (in μm): **(A)** 65; **(B)** 32.5; **(B)** (inset) 4; **(C)** 3; **(D)** 8; **(E)** 4.

We adapted the proteasome microplate fluorometric biochemical assay to cryostat sections of unfixed piglet brain, naming it the *in situ* proteasome activity assay (Figure 11). With Suc-LLVY-2R110 as substrate and the addition of 20% polyvinyl alcohol to the reaction mixture as an enzyme stabilizing and diffusion limiting agent and tissue protectant (Chayen et al., 1973; Martin et al., 1986; van Noorden and Vogels, 1989), *in situ* proteasome activity was histochemically localized in brain tissue (Figure 11). Proteasome CTL activity was present throughout the neuropil and in isolated cell bodies, putatively neurons and glia (Figures 11A,B,E). The activity was completely inhibited by MG132 (Figure 11C). In contrast to the antibody-based IHC (Figure 2), surprisingly, the proteasome CTL activity was not uniformly or homogeneously distributed throughout the neuropil; rather, it had a patchy localization in the primary somatosensory cortex, with some microregions showing higher activity than nearby regions (Figures 11A,B). Some cell bodies appeared full of proteasome activity (Figure 11E), but other cells had only very discrete inclusions of activity within the nucleus (Figure 11D) or at a perinuclear location reminiscent of the centrosome (Figures 11B,D). The number of cells with perinuclear inclusions of CTL proteasome activity was higher in the cerebral cortical gray matter compared to the subcortical white matter (Figure 11F).

## Discussion

The proteasome garners considerable attention in the cell biology of aging and neurodegeneration (Davidson and Pickering, 2023). With proteasome loss of functioning as a putative key mechanism of nervous system disease and injury throughout the lifelong spectrum, proteasome enhancement strategies are now a focus of research and development (Tripper et al., 2014; Jones et al., 2017; Trader et al., 2017; Santoro et al., 2020; El Demerdash et al., 2021; George and Tepe, 2021). Small molecule activators offer hope for clinically relevant proteasome enhancement (Tripper et al., 2014; Jones et al., 2017; Santoro et al., 2020; George and Tepe, 2021); however, much work needs completion. Some remaining hurdles include delivery approaches, testing in relevant animal models for evidence of achievable and sustainable proteasome activation, biomarkers for demonstrating and tracking proteasome effects in target tissues, and *in vivo* safety profiling of physiological effects (hemodynamics and blood chemistry) and toxicity (liver enzymes).

The experiments done here generate new knowledge on the brain proteasome. We show with antibody-based proteasome subunit localization and enzymatic activity assays the generally low brain regional specificity in several discrete gray and white matter regions; this is difficult to do in rodents, particularly for white matter. We show that neonatal piglets have a neocortical PSMB5 localization like human infants. We demonstrate an association of the proteasome with mitochondria, supported by colocalization/subcellular fractionation data. We found by a novel tissue section activity assay and localization a neocortical mosaic distribution for the proteasome. We discovered that proteasome activity in nasal mucosa biopsy can serve as a potential surrogate for proteasome activity in some brain regions, notably the hippocampus. Finally, we demonstrate the feasibility of using small molecules to activate proteasome catalytic activity in the brain with clinically relevant iv administration and no acutely observable untoward physiological effects. The latter experiment is important because, while one study showed that a brain-penetrant aryloxanyl pyrazolone derivative was therapeutic in a mouse model of ALS, brain proteasome activity was not measured during treatment (Chen et al., 2012).

Pigs are an ideal model system for the research and development of proteasome enhancement therapeutics for neurological disorders. Pigs and humans share many gross neuroanatomical and brain network complexities (Koehler et al., 2018; Simchick et al., 2019); moreover, human and pig genomes have important similarities, including synteny, gene order, small and long interspersed nuclear elements, DNA methylation, and transcriptomic RNA editing (Thomsen and Miller, 1996; Goureau et al., 2001; Schachtschneider et al., 2015; Funkhouser et al., 2017). Genome similarities extend to DNA damage and DNA repair responses engaged during cell death mechanisms in neurons exposed to oxidative stress, where pig neurons modeled human neurons better than mouse neurons in cell culture (Martin and Chang, 2018). As whole animals, transgenic pigs harboring human disease-causing mutant genes phenotypically replicate ALS (Yang et al., 2014), Huntington’s disease (Yan et al., 2018), AD (Lee et al., 2017), SMA (Lorson et al., 2011), and ataxia telangiectasia (Beraldi et al., 2015). We therefore studied the proteasome in the pig brain by evaluating the localization and biochemical activity of brain proteasomes and testing systemically administered small molecules for their ability to activate brain proteasomes.

### The proteasome appears ubiquitously uniform throughout the neonatal pig forebrain

After carefully characterizing antibodies to PSMB5 and PSMA3 for specificity by Western blotting and determining their acceptability for use in immunohistochemistry, we mapped the localization of PSMB5 in 2- to 5-day-old piglet forebrain. PSMB5 was present in virtually all neurons but only in subsets of glial cells. It had low regional specificity. In the neocortex, the PSMB5 distribution was discerning of laminar and columnar arrangements of neurons. In the hippocampus, PSMB5 was present in essentially all pyramidal neurons within each subfield. In the striatum, PSMB5 delineated virtually all neurons but not all glia. The striking neuronal positivity for PSMB5 in the normal pig brain contrasted with the moderate to low PSMB5 immunoreactivity described for the human control cerebral cortex (van Scheppingen et al., 2016). We did our own descriptive analysis of PSMB5 localization in human neocortex and found that age is likely to be an important consideration for PSMB5 immunoreactivity. A caveat of antibody-based localization and protein levels is that the information is devoid of functionality.

Proteasomes are known already for their cellular localizations in yeast and many mammalian cell types in culture (Peters et al., 1994; Rivett, 1998; Rockel et al., 2005). In human hepatoblastoma cell subnuclear fractions, proteasome α subunits were detected in the nucleoplasm but not in the nucleolus or nuclear envelop (Rockel et al., 2005). Less information is available for neurons *in situ* (Adori et al., 2005, 2006). Proteasome localization in optimally prepared brains from different animals is worthy because species differences might exist in the neuronal and glial localizations of proteasomes (Adori et al., 2006). The nuclear and cytoplasmic comparisons are also of importance because there is evidence that the nuclear proteasome lacks activity (Dang et al., 2016). The cellular localization of PSMB5 in neonatal pig neurons was conspicuous for its cytoplasmic and nuclear immunoreactivities. Much of the nuclear PSMB5 appeared to be in the nucleoplasmic matrix, but it was not seen within the nucleolus as predicted from other studies (Rockel et al., 2005). Cytoplasmic and nuclear localizations of PSMB5 were described for human neurons (Adori et al., 2006; Nakamura et al., 2006) and rat neurons (Adori et al., 2006).

There could be differential enrichments of PSMB5 in cellular compartments in pig neurons (Figure 2) compared to those shown for human neurons (Adori et al., 2005). We therefore did a pig and human brain side-by-side comparison with the same proteasome antibody, staining protocol, and imaging (Supplementary Figure 1). We found that aged adult (59–80 years old) human brain neocortical neurons show greater variation in their proteasome enrichments compared to neonatal pig (2–5 days old) neurons. For example, virtually every neuron in the neonatal pig neocortex was strongly positive for PSMB5; in contrast, many cortical neurons in the older human postcentral gyrus were negative for PSMB5 or had very low immunoreactivity, but other nearby neurons were strongly positive. In some human cortical neurons, PSMB5 immunoreactivity was enriched in the cytoplasm but low in the nucleus, while neocortical neurons in neonatal pigs universally had enrichment in the nucleus (Supplementary Figure 1). Age could be a factor in these differences, so we examined the brain of a 1-year-old infant. We found that the PSMB5 localization pattern in the human infant brain more closely resembles the neonatal piglet localization than does the brain of older humans (Supplementary Figure 1). This pilot experiment suggests brain maturation and aging have important roles in the neuronal localization of at least one proteasome subunit in the cerebral cortex.

### Proteasome types in piglet brain

The proteasome has variations in forms attributed to protein regulators. The specific forms of proteasome have different functions. The free 20S proteasome, without regulators, can selectively degrade oxidized and misfolded proteins through a facilitated unfolding mechanism in a ubiquitin- and ATP-independent manner (Pickering and Davies, 2012). Two 19S regulators can be added at either end of the 20S barrel to generate 26S-singly and -doubly capped forms, serving to limit protein targets to those that are ubiquitinated in a degradation process that is ATP-dependent (Pickering and Davies, 2012). In our in-gel activity assay, we found that the 26S-singly capped proteasome type dominated in the naïve piglet motor cortex with scant presence of the free 20S form. This proteasome composition differs from that of HeLa cells and rat tissues, where free 20S proteasome represented 40–60% of the total 20S (Tai et al., 2010). This might mean that in a naïve piglet brain with replete ATP, misfolded proteins are degraded by the ATP-dependent 26S proteasome and oxidized proteins are not formed excessively because endogenous oxidative stress is managed effectively by antioxidant systems. However, we have detected protein carbonyl accumulation in naïve piglet brain (Santos et al., 2018). More work needs to be done in piglet brain injury models to explore the presence of free 20S proteasome.

### Cytoplasmic and nuclear proteasomes both have CTL activity

An important controversy is whether cytoplasmic and nuclear proteasomes both possess protease activity. Some studies have identified proteasome proteolysis activity in the nucleus (Kim et al., 1999; Ullrich et al., 1999; Rockel et al., 2005), but another study has not (Dang et al., 2016). Proteasome subunits in the nucleus might have non-peptidase functions involving DNA repair and transcription factor-like mechanisms (Russell et al., 1999; Xie and Varshavsky, 2001). Using a validated subcellular fractionation protocol combined with proteasome CTL activity assay, we showed that both nuclear and cytoplasmic compartments possess major biochemical activities. The cytosolic proteasome is the main contributor to the total catalytic activity in crude brain tissue homogenates.

### The proteasome associates with mitochondria

More novel is our discovery that the proteasome can localize to mitochondria, where it possesses CTL activity. This finding was demonstrated microscopically and by subcellular fractionation, Western blotting for PSMB5, and an activity assay. By immunofluorescence and confocal microscopy, we found that PSMB5 partly colocalized with VDAC, cyclophilin D, and Aven in pig forebrain neurons and human neocortex. VDAC and cyclophilin D are well-known mitochondrial proteins (Martin et al., 2009, 2011). Though cytoplasmic proteasomes have recently been associated with mitochondrial protein quality control in a process in yeast called mitochondria-associated degradation (Rödl and Herrmann, 2023), a mitochondrial localization of proteasome has not been shown before. The proteasome could be tethered to the outer mitochondrial membrane, like DNA methyltransferase-1 (Wong et al., 2013), potentially associated with the translocator of the outer mitochondrial membrane (Bertolin et al., 2013), or within the intermembrane space, inner membrane, or mitochondrial matrix. Sub-mitochondrial fractionation and intact mitochondria-protease digestion experiments need to be done to decipher how the proteasome is associated with mitochondria. Intriguingly, the mitochondrial associated proteasome has CTL activity. It contributes about 15–20% of the total activity of crude homogenate in the cerebral cortex.

A mitochondrial-associated proteasome would be a complement to known mitochondrial ATPases associated with diverse cellular activities (AAA+) proteases found within the intermembrane space (e.g., iAAA), inner membrane (e.g., SPG7/paraplegin), and matrix (e.g., LONP1 and ClpXP) (Feng et al., 2021). The AAA+ mitochondrial proteins have many non-peptolytic functions, in addition to protease activities that degrade preferential mitochondrial protein targets that are misfolded or oxidatively damaged. For example, LONP1 functions in mitochondrial DNA replication and the degradation of oxidatively damaged aconitase (Bota and Davies, 2002). SPG7/paraplegin may be a critical component of the mitochondrial permeability transition pore (Shanmughapriya et al., 2015) and assemble mitochondrial ribosomes, in addition to proteolyzing damaged cytochrome c oxidase subunit 1 (Almontashiri et al., 2014). Interestingly, there are several non-mitochondrial cytoplasmic proteins in aberrantly misfolded, oxidized, cleaved, and otherwise pathological states (e.g., SOD1, αSyn, β-amyloid) that gain access to mitochondria to induce mitochondriopathy (Martin et al., 2006, 2007; Ruan et al., 2017). These abnormal proteins might not be substrates for highly specialized and dedicated mitochondrial AAA+ proteases. A mitochondrial-associated proteasome with broad peptidolytic capacity could provide an adaptive advantage to cells, and possibly individuals, for degrading xenoproteins binding to the surface of mitochondria or anomalously gaining access to mitochondria with potential toxicities, such as SOD1, αSyn, and β-amyloid. However, caution is warranted regarding evidence for an intrinsic mitochondrial proteasome until a comparative protease substrate profile is done with mitochondrial AAA+ proteases. While Suc-LLVY-AMC is a preferred substrate of PSMB5, and this proteasome subunit was detected in lysates of pure mitochondria, there could be some, albeit non-preferred, substrate overlap with ClpXP (Arribas and Castaño, 1993).

### Our novel *in situ* proteasome histochemical assay reveals a new proteasome landscape in the cerebral cortex

We adapted the standard homogenate-based microplate fluorometric assay for proteasome CTL activity to an unfixed brain cryostat section assay. This assay is novel. The Y-2R110 reaction product formed by CTL cleavage of Suc-LLVY-2R110, stabilized by polyvinyl alcohol, was completely inhibitable by MG132. With this assay, proteasome CTL activity was visualized in cells and in the neuropil. This histological-based assay yielded different data compared to the antibody-based IHC that gave the impression of brain regional uniformity and generalized enrichment in neurons. However, the histochemical proteasome activity assay suggests a cellular heterogeneity and proteasome complexity hitherto undisclosed by homogenate- and antibody-based assays.

The histochemical activity assay was used on the piglet somatosensory cortex. It revealed that neurons and glial cells do not appear to be enriched similarly in proteasome CTL activity. It also directly showed nuclear proteasome catalytic activity. Within the neuropil, there was patchiness in catalytic activity, giving the impression of a functional mosaic of the proteasome in the cerebral cortex. We also found a distinguished proteasome compartment residing in a perinuclear position. We observed it with difficulty by standard immunolocalization, but our new *in situ* histochemical activity assay particularly highlighted this organelle because of the striking CTL activity. This perinuclear proteasome satellite might be the centrosome-associated proteasome seen structurally and biochemically in cultured HEK293 and HeLa cells (Wigley et al., 1999; Fabunmi et al., 2000). The structure was more common in cortical gray matter than in subcortical white matter, though it was not seen in every cell. To the best of our knowledge, this structure has not been described before in the brain. It might correspond to the aggresome described in yeast (Enenkel et al., 2022), but its robust visualization in naïve piglets suggests that this structure is not pathological and was formed only in response to an overload of misfolded proteins. If this perinuclear CTL activity-enriched proteasome satellite seen in the pig brain is centriole-related, then the centriole pair would be embedded in a matrix enriched in pericentriolar material 1 protein (PCM1) (Dammermann and Merdes, 2002). We have discovered that PCM1 is particularly sensitive to carbonyl oxidation in neonatal piglets with hypoxia–ischemia and treatment with hypothermia (El Demerdash et al., 2021) and, thus, the centriole might destabilize in neonatal brain injury. Because the centriole functions as a site for microtubule assembly for neurites and dendrites (Yu et al., 1993), PCM1 oxidation could be a pathological mechanism for dendritic or oligodendrocyte neurite beading and obliteration that we have seen in piglet encephalopathy (Lee et al., 2021).

### There is a brain neuropil proteasome

We studied the not-often-discussed presence of the proteasome in the neuropil throughout the piglet forebrain. We observed this by immunohistochemistry and with our novel *in situ* enzyme histochemical assay. The catalytically active proteasome in the neuropil was completely inhibited by MG132. This brain parenchymal neuropil proteasome distribution appeared elegantly macro-compartmentalized as a mosaic in pig and human neocortex. A neuropil proteasome is interesting because it could be mitochondrial associated, as supported by the data shown here, and synaptic. A synaptic proteasome has been suggested to participate in endosomal uptake of glutamate receptors (Bingol and Schuman, 2004), activity-dependent structural plasticity (Bingol and Schuman, 2006), and long-term potentiation (Fonseca et al., 2006; Dong et al., 2008). Neuropil proteasomes could also be glial-associated (Tydlacka et al., 2008). It is unknown whether pharmacologically forced activation of proteasome activity would have deleterious effects on these proteasomal functions.

### Activation of the brain proteasome is achievable with systemic administration of small molecules

We have identified an apparently safe drug combination to increase CTL proteasome activity generally throughout the neonatal gyrencephalic brain with systemic treatment. In piglets treated intravenously with boluses of pyrazolone derivative and chlorpromazine cocktail over 24 h, brain proteasome CTL activity was modestly increased. Western blotting for PSMB5 indicated that upregulation of PSMB5 levels did not account for this catalytic increase (Supplementary Figure 2). Pyrazolone analogs are known to bind and activate the proteasome in cell-free and cell systems (Tripper et al., 2014) and showed promise in a mouse model of ALS involving mutant SOD1 proteinopathy. Chlorpromazine, a clinical drug used safely for over 5 decades, also activates the proteasome in cell systems (Jones et al., 2017). In piglets treated with CPZ, the sedation and motor effects were dose-limiting. Proteasome activation with PYR/CPZ seems possible for the cerebral cortex and deep forebrain, gray matter, and white matter. Importantly, these regions are disease- and injury-vulnerable in many human neurological conditions in which proteasome insufficiency is pathogenically relevant. Many future experiments are needed, including additional PYR dose ramping, drug effects on the other two catalytic activities of the proteasome, pharmacokinetics and brain half-life determinations, brain effect durations, and long-term dosing with toxicological and behavioral profiling for safety.

### A nasal marker for brain proteasome activity

We discovered that proteasome functional activity in some brain regions correlates with proteasome activity in the nasal mucosa. Hippocampal and nasal mucosal activities are notably correlated. Even the protein levels of PSMB5 in the nasal mucosa matched those of several different brain regions. This finding could be relevant to CNS drug therapy development. A substantial impasse in CNS drug discovery for human brain and spinal cord injury and disease is demonstrating bioavailability and target engagement in the CNS of patients (Kingwell, 2011). If the nasal mucosa epithelial lining can serve as a surrogate for CNS proteasome activity, then this could advance the preclinical search for proteasome-related drug development of therapeutics for neurological disorders related to proteinopathy.

## Conclusion

We studied the brain proteasome in pigs because this species is useful for modeling proteinopathy and encephalopathy involving degeneration of neurons and glia. Of the three different catalytic activities of the proteasome, we focused on the CTL activity. The PSMB5 subunit was ubiquitously present in the pig brain. In naïve piglets, proteasome CTL activity levels were relatively uniform in many different brain regions. By rigorous subcellular fractionation, proteasome CTL activity was present in cytosolic, nuclear, and pure mitochondrial fractions. All fractions possessed CTL activity. PSMB5 is partly colocalized with mitochondrial proteins in the pig and human neocortex. In-gel activity determinations found the 26S-single capped the prominent type of proteasome in the pig cerebral cortex. A new histochemical proteasome activity assay revealed in the cerebral cortex a novel proteasome landscape. A cocktail of pyrazolone derivative and chlorpromazine can possibly increase brain CTL activity with iv treatment. More dosing strategies need to be tested. Overall, this study is pertinent to further elucidating mechanisms of encephalopathy in the neonatal and adult nervous systems and the development of small molecule therapeutics for alleviating proteinopathy in brain disorders and diseases.

## Data availability statement

The raw data supporting the conclusions of this article will be made available by the authors, without undue reservation.

## Ethics statement

This study used human postmortem autopsy tissue. All autopsies had approved consent. The protocol for use of human autopsy tissue was reviewed and approved by the JHMI-IRB (application number NO:02–09024-04e). The use of human tissues was also approved by the JHMI-Office of Health, Safety and Environment (JHU registration B1011021110). The studies were conducted in accordance with the local legislation and institutional requirements. Written informed consent for participation was not required from the participants or the participants’ legal guardians/next of kin in accordance with the national legislation and institutional.

## Author contributions

AA: Data curation, Formal analysis, Methodology, Writing – review & editing. MC: Data curation, Formal analysis, Investigation, Writing – review & editing. ND: Data curation, Formal analysis, Writing – review & editing. CJ: Investigation, Methodology, Writing – review & editing. DP: Data curation, Formal analysis, Writing – review & editing. JL: Conceptualization, Data curation, Formal analysis, Funding acquisition, Investigation, Methodology, Project administration, Resources, Writing – review & editing. LM: Conceptualization, Data curation, Formal analysis, Funding acquisition, Investigation, Methodology, Project administration, Supervision, Writing – original draft, Writing – review & editing.
