# Supplementary figures and images for: Proteasome localization and activity in pig brain and in vivo small molecule screening for activators
Source: Front Cell Neurosci. 2024 Feb 26;18:1353542. doi: 10.3389/fncel.2024.1353542 (PMC10925635; doi:10.3389/fncel.2024.1353542)

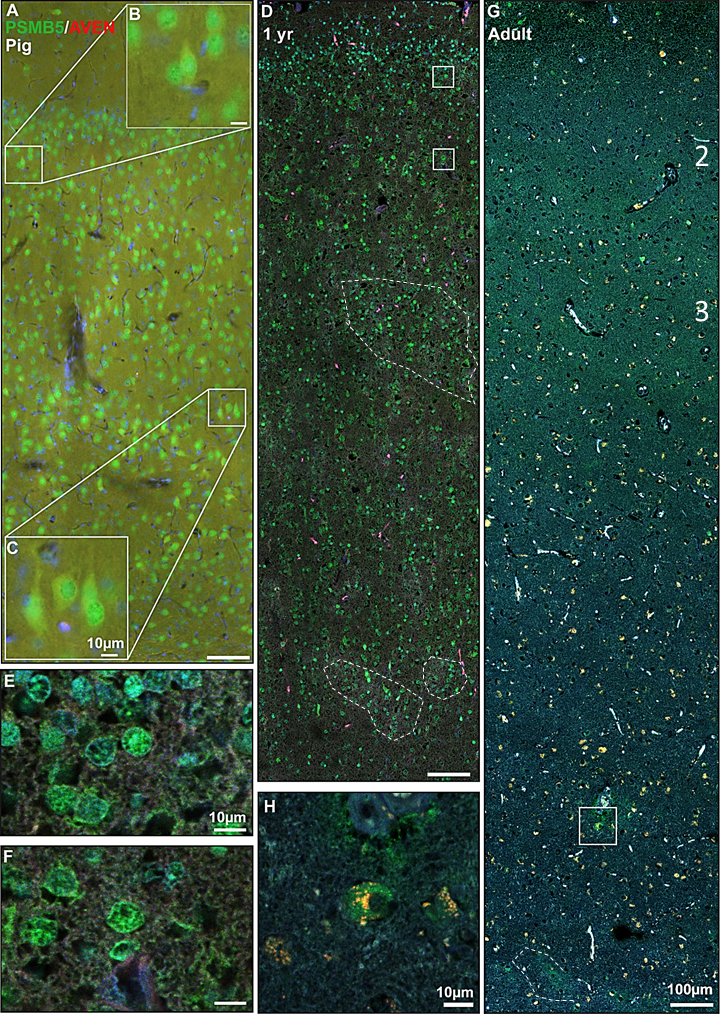

Supplement: Supplementary file 1 [file Image_1.TIF]

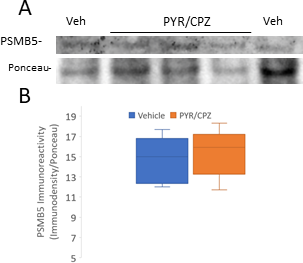

Supplement: Supplementary file 2 [file Image_2.TIF]
